# Epigenetic regulation of *Atrophin1* by lysine-specific demethylase 1 is required for cortical progenitor maintenance

**DOI:** 10.1038/ncomms6815

**Published:** 2014-12-18

**Authors:** Feng Zhang, Dan Xu, Ling Yuan, Yiming Sun, Zhiheng Xu

**Affiliations:** 1 State Key Laboratory of Molecular Developmental Biology, Institute of Genetics and Developmental Biology, Chinese Academy of Sciences, 1 West Beichen Road, Beijing 100101, China; 2 University of Chinese Academy of Sciences, Beijing 100101, China; 3 Parkinson’s Disease Center, Beijing Institute for Brain Disorders, Beijing 100101, China

## Abstract

Lysine-specific demethylase 1 (LSD1) is involved in gene regulation and development; however, its precise function, molecular targets and underlying mechanisms during development are poorly understood. Here we show that LSD1 is required for neuronal progenitor cell (NPC) maintenance during cortical development. A ChIP-seq analysis identified a LSD1-binding site (LBAL) downstream of *Atrophin1* (*ATN1*). Surprisingly, tranylcypromine (LSD1 inhibitor) treatment increased H3K4 methylation at LBAL, leading to *ATN1* repression and NPC differentiation. Knockdown of LSD1 and ATN1 phenocopied each other in inducing NPC premature differentiation and depletion, which could be rescued by ATN1 overexpression, suggesting that LSD1 controls NPC differentiation via regulation of *ATN1* methylation status and expression. The involvement of LSD1 in ATN1 expression and NPC maintenance were confirmed in knockout mice. These findings hint at the potential application for the clinical drug, tranylcypromine, in the prevention and/or treatment of *ATN1*-associated degenerative disease, dentatorubral-pallidoluysian atrophy.

## Abstract (web summary)

Histone modification is critical for gene expression regulation during development. Here, the authors show that the demethylase LSD1 and its target gene *ATN1* are responsible for maintenance of neural progenitor cells during mouse cortical development.

## Graphical abstract

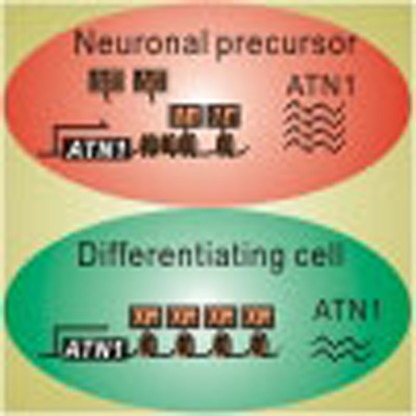

Epigenetic regulation of gene expression plays a critical role in many biological processes, including cell reprogramming, development, tumorigenesis and ageing^1^,^2^,^3^,^4^,^5^. Histone methylation is one type of epigenetic modification which, when perturbed, leads to various diseases^6^,^7^,^8^.

The development of the mammalian neocortex requires a balance between self-renewal and differentiation of neural progenitor/precursor cells (NPCs, predominantly radial glial cells) followed by neuronal migration and maturation^9^,^10^,^11^,^12^. Lysine-specific demethylase 1 (LSD1), a histone demethylase for mono- or dimethylated H3K4 and H3K9 (ref. 13), has been indicated in different aspects of brain development, including pituitary development^14^, cortical neuron migration^15^ and adult NPC proliferation^16^. LSD1 is also required for embryonic stem cell differentiation^17^. In addition, LSD1 was recently identified as a target of the microRNA miR-137; overexpression of miR-137 induced premature differentiation and migration of immature neurons^18^. Thus, although this implicates that LSD1 may be involved in coordinating NPC proliferation versus differentiation, detailed information on its molecular targets and mechanisms of action is still lacking.

Dentatorubral-pallidoluysian atrophy (DRPLA) is an autosomal dominant neuronal degenerative disease characterized by ataxia, choreoathetosis and cognitive dysfunction, including dementia, resulting from polyglutamine (polyQ) expansion in *Atrophin1* (*ATN1*)^19^,^20^,^21^. ATN1 interacts with, and modulates the activity of, the transcriptional repressor TLX^22^. *ATN1* knockout mice are viable^23^, but the function of ATN1 in brain development has not yet been examined.

The present study investigated the role of LSD1 in the neocortex to determine whether epigenetic changes in gene expression during embryonic development affect progenitor cell maintenance. We provided evidence here that *ANT1* is a direct target of LSD1 and that knockdown or knockout of LSD1 leads to the downregulation of *ATN1* expression. Knockdown of either LSD1 or ATN1 induces significant premature differentiation and depletion of NPCs, and it can be rescued by *ATN1* expression. Moreover, the clinical LSD1 inhibitor, tranylcypromine, suppressed *ATN1* expression, suggesting that it could be applied to the treatment of adult-onset DRPLA.

## Results

### LSD1 depletion results in premature NPC differentiation

**Figure 1:**
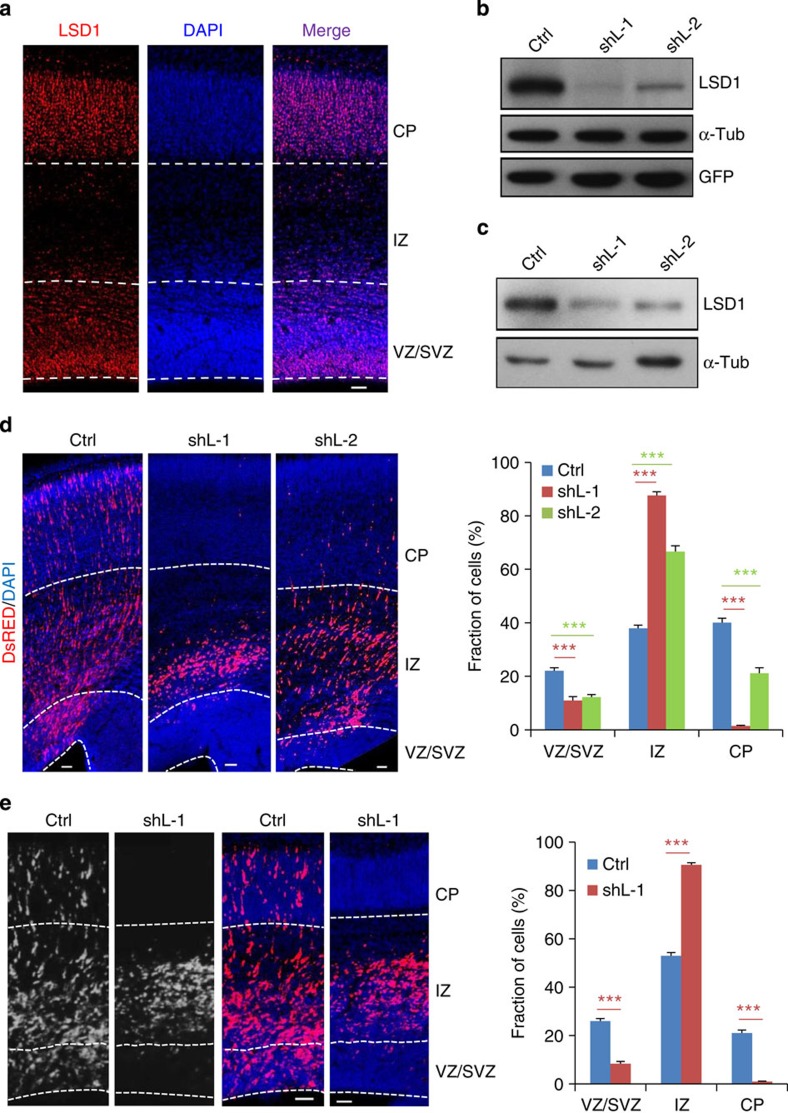
LSD1 knockdown leads to altered cell distribution in the cortex. (**a**) Coronal sections from E18.5 rat cortex stained with 4′,6-diamidino-2-phenylindole (DAPI) to label nuclei (blue) and an antibody against LSD1 (red). (**b**,**c**) Endogenous and overexpressed LSD1 are efficiently knocked down by LSD1 shRNA (shL). (**b**) pEGFP-N1-LSD1 was co-transfected with a control shRNA (shCtrl) or shL into HEK293 cells; 24 h later, cell lysates were analysed by immunoblotting with anti-LSD1 antibody, with α-tubulin serving as a loading control and GFP as a control for transfection efficiency. (**c**) Ctrl or shL was transfected into N2A cells; 48 h later, DsRed^+^ cells were collected by fluorescence-activated cell sorting and analysed by immunoblotting with anti-LSD1 antibody. (**d**) Coronal sections of rat brains electroporated *in utero* with Ctrl or shL at E16.5 and examined at E20.5. Right panel: quantification of DsRed^+^ cell distribution in the cortex. Data represent mean±s.e.m. ***P<0.001 (Ctrl, *n*=26/8; shL-1, *n*=24/7; shL-2, *n*=10/3; one-way analysis of variance). (**e**) Cortex electroporated at E15.5 and analysed at E18.5. Data represent mean±s.e.m. ***P<0.001 (Ctrl, *n*=21/7; shL-1, *n*=27/9; Student’s *t*-test). *n*, number of slices from different brains. Scale bars, 50 μm.

The expression of LSD1 in the developing rat brain was first examined. LSD1 immunoreactivity was observed in the nucleus in all layers of the cortex, including the proliferative ventricular and subventricular zones (VZ and SVZ, respectively), the intermediate zone (IZ) through which newly born neurons migrate and the nascent cortical plate (CP) (Fig. 1a and Supplementary Fig. 1a). Two short hairpin RNA (shRNA)/DsRed constructs were found to be highly efficient in knocking down both overexpressed and endogenous LSD1 (Figs 1b,c and 3g, and Supplementary Fig. 6a). These shRNA constructs were electroporated into rat cortex via *in utero* electroporation (IUE) at embryonic day 16.5 (E16.5) and the brains were examined 4 days later (E20.5). In control embryos transfected with a control shRNA/DsRed construct, DsRed^+^ cells were distributed in all the layers (Fig. 1d). In contrast, on LSD1 knockdown most of the transfected cells were found in the IZ, with very few in the CP, consistent with a previous report that LSD1 depletion inhibits neuronal migration^15^. However, much fewer cells were observed in the VZ and SVZ compared with controls (Fig. 1d). Cortical neurons arise on a chronological schedule with subtypes of neurons emerging in a defined order^24^,^25^; therefore, we performed the IUE experiment from E15.5 to E18.5 similar to what we did previously^26^. Similar results of NPC depletion were obtained (Fig. 1e), indicating that LSD1 may regulate NPC development at different stages.

We examined whether the loss of NPC induced by LSD1 knockdown is due to increased cell death, by staining the brain sections with the activated form of caspase 3, and found no apparent increase of cell death (Supplementary Fig. 1b). In addition, co-transfection of a shRNA-resistant LSD1 construct rescued the depletion of NPCs in the VZ and SVZ (Supplementary Fig. 2a–c), thereby excluding the possibility of shRNA off-target effects.

**Figure 2:**
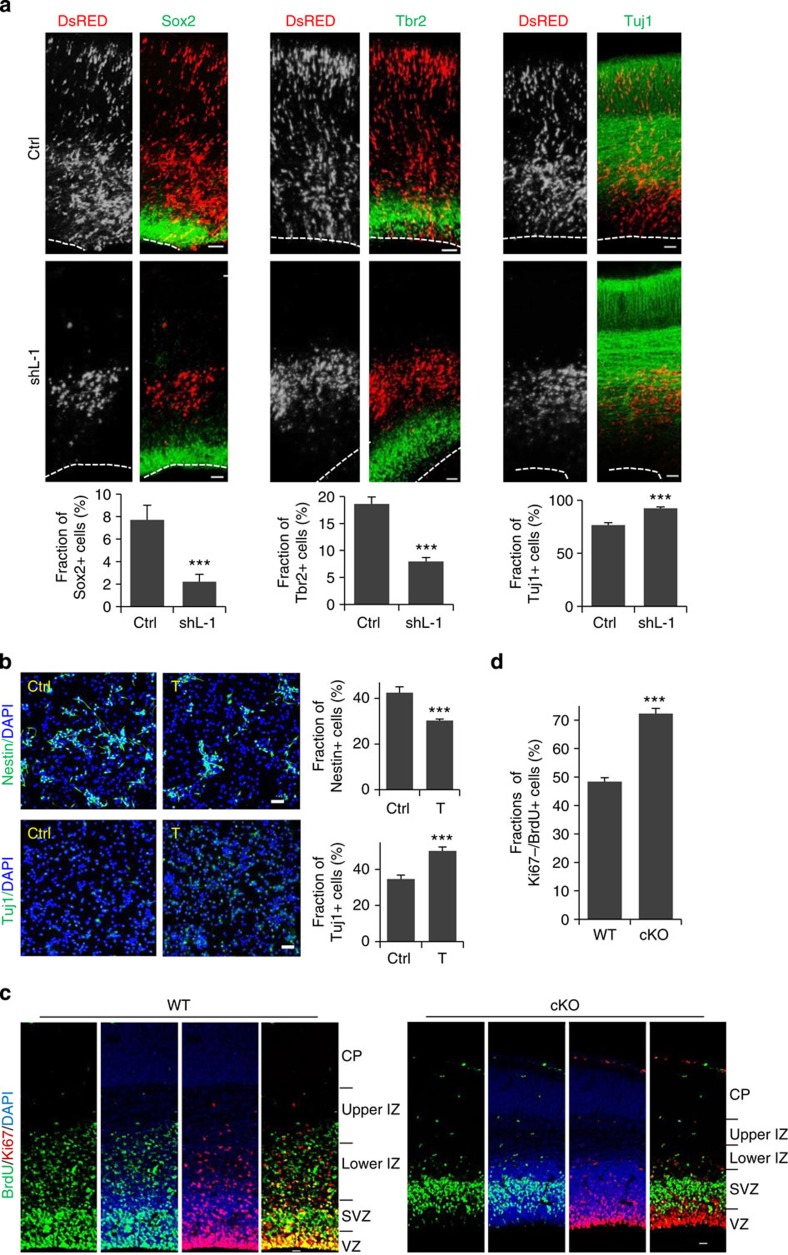
LSD1 depletion leads to premature differentiation of NPCs. (**a**) LSD1 knockdown leads to premature differentiation of NPCs. Coronal sections of cortices electroporated with shCtrl or shL-1 at E15.5 were examined at E18.5 by staining with antibodies against Sox2, Tbr2, or Tuj1; the quantitative analysis is shown in the lower panels. Data represent mean±s.e.m. ***P<0.001, *t*- test. Sox2 (Ctrl, *n*=7/7; shL-1, *n*=10/10); Tbr2 (Ctrl, *n*=7/7; shL-1, *n*=10/10); Tuj1 (Ctrl, *n*=7/7; shL-1, *n*=8/8). *n*, number of slices from different brains. (**b**) E16.5 rat cortices were dissociated and cultured in DMEM/B27/bFGF/EGF for 24 h, then treated with saline (Ctrl) or tranylcypromine (T, 10 μM) for 24 h. After fixation, cells were stained for Nestin and Tuj1. Quantitative analyses are shown in the lower panels. Data represent mean±s.e.m. ***P<0.001 (Student’s t-test, >4,000 cells were counted for each group). (**c**,**d**) Coronal cortical sections from E16.5 wild-type or brain-specific LSD1-cKO mice injected with BrdU (100 μg kg^−1^) at E15.5 were immunostained with BrdU and Ki67 antibodies (**c**). Nuclei were labelled with 4′,6-diamidino-2-phenylindole (DAPI). Scale bar, 20 μm. The fractions of BrdU^+^/Ki67^−^ cells were quantified in **d**. Data represent means±s.e.m. ***P<0.001 (WT: *n*=9/3; cKO: *n*=9/3; Student’s *t*-test). *n*, number of slices from different brains.

To determine whether the depletion of transfected cells in the proliferative zones corresponded to cell fate changes, cells transfected with LSD1 shRNA/DsRed from E15.5 to 18.5 were examined for the expression of the progenitor markers Sox2 and Tbr2—which label apical progenitors/radial glial cells of the VZ and intermediate/basal progenitors of the SVZ, respectively—as well as the early neuronal marker Tuj1. LSD1 knockdown resulted in substantial decrease of Sox2^+^ and Tbr2^+^ cells, and a corresponding increase of Tuj1^+^ cells, and these could be rescued by co-expression of human LSD1 (Fig. 2a and Supplementary Fig. 2d). These results indicate that the loss of LSD1 in NPCs induces their premature differentiation into neurons.

To determine whether the observed effect was due to the demethylase activity of LSD1, cortical progenitor cells dissociated at E16.5 were treated with the LSD1 inhibitor tranylcypromine^27^ and examined 24 h later by staining cells with the progenitor cell marker Nestin and the differentiation marker Tuj1. Inhibition of LSD1 activity induced the differentiation of NPCs, as indicated by the significantly increased number of Tuj1^+^ cells and concomitant decrease in Nestin^+^ cells (Fig. 2b).

**Figure 3:**
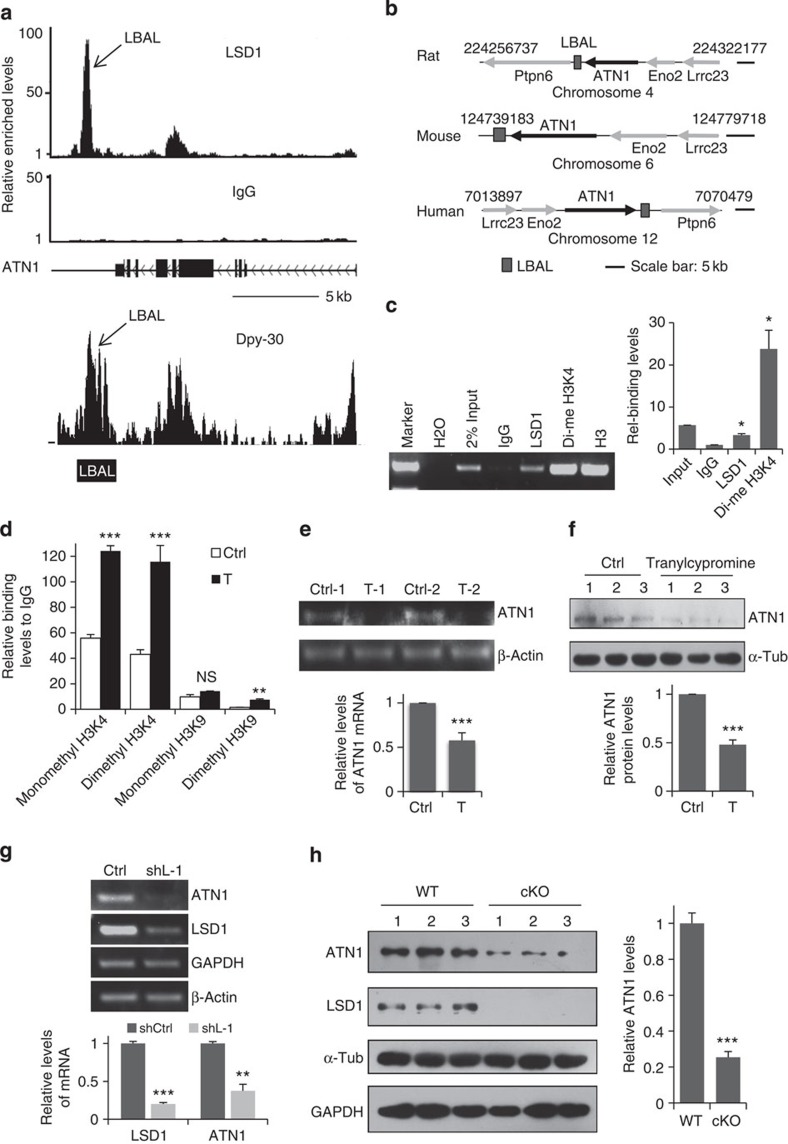
LSD1 binds LBAL and regulates dimethyl H3K4 level and *ATN1* expression. (**a**) LSD1 binds to the LBAL site downstream of *ATN1* identified by ChIP-seq (upper panel). Dyp-30 also binds to the LBAL site, according to Dpy-30 ChIP-seq results deposited in the GEO database under GSE26136 (lower panel). Arrow: LSD1- and Dpy-30-binding peaks in similar location with LBAL site. Scale bar, 5 kb. (**b**) LBAL sites are conserved in human, mouse and rat. (**c**) Both LSD1 and dimethyl H3K4 bind to LBAL site, as determined by ChIP of E16.5 rat brain followed by qPCR. *P=0.029 (LSD1, *n*=3); *P=0.047 (dimethyl H3K4, *n*=3; paired *t*-test). (**d**) LSD1 inhibitor increases H3K4 dimethylation and monomethylation at LBAL. E16.5 rat brains were dissociated and cultured in DMEM/N2/bFGF for 12 h, then treated with saline (Ctrl) or tranylcypromine (T, 10 μM) for 8 h, followed by ChIP with IgG, or corresponding antibodies as indicated. ***P<0.001, **P<0.01, NS, not significant. (*n*=3, except for dimethyl H3K4, *n*=6; t test). (**e**,**f**) LSD1 inhibition suppresses *ATN1* expression. NPCs were treated as in **d** for 12 h. (**e**) *ATN1* mRNA analysed by qPCR. ***P=0.0002 (*n*=14). (**f**) Protein levels determined by western blotting. ***P=0.0001 (*n*=6; paired *t*-test). (**g**) Rat brains were electroporated with shCtrl or shL-1 at E16.5 and DsRED^+^ cells were collected by fluorescence-activated cell sorting at E19.5. *LSD1* and *ATN1* mRNA levels in these cells were analysed by qPCR. **P=0.002, ***P<0.001, (*n*=3; *t*-test). (**h**) Protein levels in E14.5 wild-type or LSD1 cKO cortices were determined by western blotting. ***P=0.0003 (*n*=3; *t*-test). All data represent mean±s.e.m.

To further confirm the role of LSD1 in neuronal differentiation, *LSD1*^*flox/flox*^ mice were crossed with Nestin-*Cre* mice to generate the *LSD1* brain-specific conditional knockout (*LSD1* cKO) mice. Loss of LSD1 expression in cKO mouse brains was verified by immunostaining and western blotting (Fig. 3h and Supplementary Fig. 3a). cKO mice have smaller brains and thinner cortices compared with their wild-type littermates (Supplementary Fig. 3a). E15.5 wild-type or *LSD1* cKO mice were then injected with 5-bromodeoxyuridine (BrdU) and analysed 24 h later. Similar to that of LSD1 knockdown, BrdU^+^ cells in the VZ were depleted in the *LSD1* cKO brains (Fig. 2c), with most BrdU^+^ cells localized in the SVZ and much less cells reaching the IZ, in contrast to their wild-type littermates (Supplementary Fig. 3b). More importantly, the fraction of Ki67^−^/BrdU^+^ cells increased significantly in the *LSD1* cKO brains, indicating that more *LSD1* cKO cells underwent cell cycle exit and premature differentiation (Fig. 2c,d). Probably due to the premature differentiation, the number of cells incorporated with BrdU decreased substantially in *LSD1* cKO mice (Supplementary Fig. 3c). We also dissected NPCs from E14.5 mice, cultured for 48 h and examined the Nestin and Tuj1 markers. Similar to LSD1 inhibitor treatment, knockout of *LSD1* induced differentiation of NPCs, as revealed by the reduction of Nestin^+^ cells and increase of Tuj1^+^ cells (Supplementary Fig. 3d,e). Taken together, our results demonstrate that LSD1 is required for the maintenance of NPCs during cortical development.

### LSD1 positively regulates *ATN1* expression

To further investigate the mechanism by which LSD1 maintains the progenitor pool, chromatin immunoprecipitation sequencing (ChIP-seq) was performed in E16.5 brain homogenates to identify potential targets of LSD1. Genes encoding histones were the most highly represented among the potential targets (Supplementary Table 1), consistent with previous reports and the known function of LSD1 (ref. 28). A novel candidate gene was *ATN1*. Interestingly, the analysis revealed a putative LSD1-binding site downstream of the *ATN1* coding region, as opposed to within the promoter (Fig. 3a and Supplementary Fig. 4). A homology search indicated that this sequence, which was named LBAL (LSD1-binding site at the *ATN1* locus), is conserved in rodents and humans (Fig. 3b and Supplementary Fig. 5). Intriguingly, ChIP-seq results by a previous report indicate that the binding site of the SET1/MLL histone methyltransferase complex subunit, Dpy-30, overlaps with LBAL (Fig. 3a)^29^. The binding of LSD1 and K4-dimethylated histone H3 (dimethyl H3K4) to LBAL was confirmed by ChIP–quantitative PCR (qPCR), which showed enrichment by 3.3- and 23.8-fold, respectively, relative to the IgG control (Fig. 3c). In addition, treatment of cultured cortical progenitors for 8 h with tranylcypromine increased the levels of both dimethyl and monomethyl H3K4 at LBAL without apparently altering the H3K9 monomethylation at LBAL (Fig. 3d), suggesting that the demethylase activity of LSD1 is normally required to suppress H3K4 methylation at this site. Moreover, both messenger RNA and protein expression of *ATN1* was suppressed by tranylcypromine treatment (Fig. 3e,f), corresponding to the observed premature differentiation of NPCs (Fig. 2b). Although the dimethyl (not monomethyl) H3K9 level at LBAL was also enhanced by tranylcypromine treatment, much less histone methylation modification occurred in H3K9 than in H3K4, suggesting that LBAL site is mostly occupied and regulated by H3K4 methylation modification and not H3K9 methylation (Fig. 3d).

To confirm that LSD1 regulated *ATN1* expression *in vivo*, rat brains were electroporated with shLSD1 at E16.5, and 3 days later those DsRED^+^ cells were collected through fluorescence-activated cell sorting. Consistently, the qPCR results showed the efficient knockdown of *LSD1* by shLSD1 and significant downregulation of *ATN1* as well (Fig. 3g). This was also confirmed by immunostaining of those cells transfected with shLSD1 in the VZ with both LSD1 and ATN1 antibodies (Supplementary Fig. 6a,b). More importantly, we detected significantly lower ATN1 protein levels in E14.5 cortices of brain-specific *LSD1* cKO mice (Fig. 3h). Decreased *ATN1* expression was also confirmed in the NPCs residing in the VZ of *LSD1* cKO mice cortex by immunostaining (Supplementary Fig. 6c,d). All these *in vitro* and *in vivo* results demonstrate that *ATN1* expression is positively regulated by LSD1. This finding was unexpected, as H3K4 dimethylation is typically associated with transcriptional activation^16^; thus, LSD1 demethylase activity would be predicted to suppress *ATN1* expression, while tranylcypromine treatment or LSD1 knockout should have the opposite effect.

Given that LSD1 binds to the LBAL site downstream of *ATN1* locus and positively regulates *ATN1* expression, we investigated whether the regulation of *ATN1* expression by LSD1 is through LBAL. The LBAL sequence (1.98 kb) was cloned upstream of the enhanced green fluorescent protein (eGFP) reporter in the pCMS-eGFP backbone (pCMS-eGFP-LBAL), becoming a downstream insertion on vector linearization (Supplementary Figs 7a and 4). Interestingly, the presence of the LBAL downstream of GFP dramatically enhanced the expression of GFP (Supplementary Fig. 7c). Tranylcypromine treatment substantially suppressed GFP expression in cells transfected with pCMS-eGFP-LBAL but not those with pCMS-eGFP (Supplementary Fig. 7b,c), and increased the dimethyl H3K4 level at the LBAL site in the linearized plasmids (Supplementary Fig. 7d). Thus, our results suggest that LSD1 regulates *ATN1* expression through the modulation of LBAL methylation status. In addition, the LBAL site may play a role as an enhancer for gene expression and its activity is likely to be regulated by increased H3K4 methylation level.

### *ATN1* expression is associated with H3K4 methylation levels at LBAL

**Figure 4:**
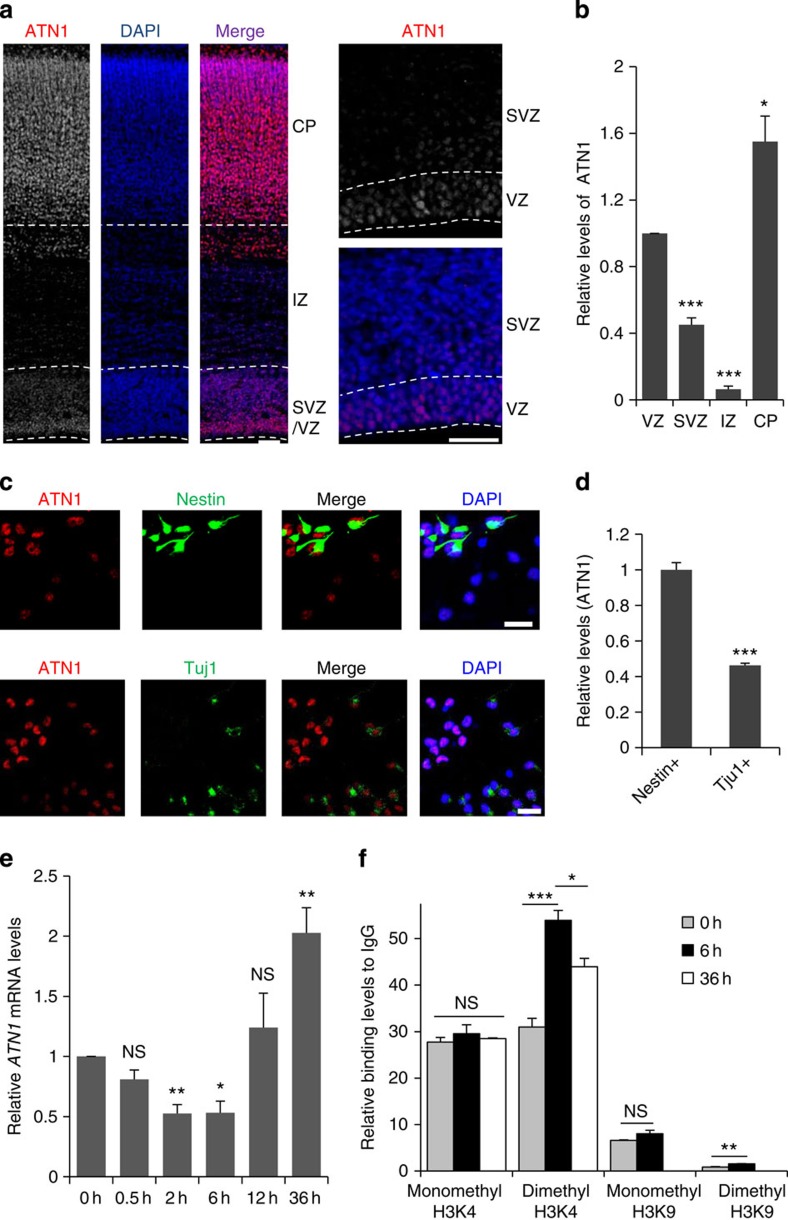
*ATN1* expression is downregulated during NPC differentiation. (**a**) E20.5 rat coronal cortical sections stained with ATN1 antibody and 4′,6-diamidino-2-phenylindole (DAPI). Right panels show high-magnification view of the VZ and SVZ. Scale bar, 50 μm. (**b**) Quantification of ATN1 levels (normalized to DAPI) in the VZ, SVZ, IZ and CP. ***P<0.001, *P=0.015, (*n*=6/3; paired *t*-test). *n*, number of sections from different brains. (**c**) E16.5 rat brains were dissociated and cultured in DMEM/B27/bFGF/EGF/P/S for 48 h, fixed, then immunostained for ATN1 and Nestin (upper panel) or Tuj1 (lower panel) and DAPI. Scale bar, 20 μm. (**d**) Quantification of ATN1 levels in Nestin^+^ and Tuj1^+^ cells. ***P<0.001 (Nestin, *n*=50; Tuj1, *n*=50; Student’s *t*-test). (**e**) Quantification of *ATN1* mRNA levels in neurospheres at time indicated after bFGF and EGF withdrawal by real-time PCR. *P=0.017, **P=0.0076 (2 h), **P=0.0081 (36 h). (0.5 h, *n*=4; 2 h, *n*=4; 6 h, *n*=4; 12 h, *n*=7; 36 h, *n*=5; paired *t*-test). NS, not significant. (**f**) Neurospheres from dissociated E16.5 rat brains were cultured in DMEM/B27/bFGF/EGF for 36 h before bFGF and EGF withdrawal as in **e**; 6 or 36 h later, different histone modification levels at LBAL were analysed as in Fig. 3d. *P=0.024, **P=0.0028, ***P<0.001 (*n*=3; except for dimethyl H3K4 in 6 h, *n*=6; one-way analysis of variance). All data are means±s.e.m.

The knockdown of the SET1/MLL histone methyltransferase complex subunits, RbBP5 and Dpy-30, has been reported to direct the differentiation potential of embryonic stem cells along the neural lineage^29^, with concomitant defects in gene repression, including *ATN1* (Supplementary Fig. 8). In addition, we have shown above that LSD1 regulates *ATN1* expression and NPC differentiation. Thus, we hypothesized that LSD1 could maintain a progenitor state in NPCs by antagonizing methyltransferase activity to regulate *ATN1* expression. To test this, the expression of *ATN1* in the embryonic brain was examined. ATN1 immunoreactivity was high relative to the nuclear (4′,6-diamidino-2-phenylindole) signal in the apical progenitor cells within the VZ, lower in the intermediate/basal progenitors of the SVZ, the lowest in the IZ and higher in neurons in the CP (Fig. 4a,b). Similarly, *ATN1* expression in Nestin^+^-cultured NPCs was substantially higher than in Tuj1^+^-differentiated neurons (Fig. 4c,d). These results imply that a downregulation of *ATN1* expression is required for differentiation to occur, after which the levels again increase. To test this, basic fibroblast growth factor (bFGF) and epidermal growth factor (EGF) were withdrawn from the culture medium to induce differentiation of NPCs in a neurosphere culture. Within 2–6 h of growth factor withdrawal, *ATN1* mRNA levels decreased, followed by an increase at 12–36 h, a trend similar to what was observed *in vivo* (Fig. 4e).

To test whether the histone methylation levels at LBAL site was associated with the regulation of *ATN1* expression during NPC differentiation, neurosphere cultures were subjected to growth factor withdrawal, to induce differentiation, and different histone methylation modification levels at LBAL were evaluated by ChIP–qPCR. As shown in Fig. 4f, H3K4 dimethylation levels at LBAL increased very significantly at 6 h after growth factor withdrawal but dropped relatively at 36 h, corresponding to the expression of *ATN1* (Fig. 4e). Meanwhile, the H3K4 and H3K9 monomethylation levels were not apparently changed. Although the dimethyl H3K9 levels increased 6 h after growth factor withdrawal, similar to tranylcypromine-treated NPCs (Fig. 3d), it’s enrichment was also much lower than that of H3K4.

To investigate the role of LBAL in the regulation of *ATN1* expression *in vivo*, linerated pCMS-eGFP or pCMS-eGFP-LBAL together with control or shLSD1 were introduced into NPCs of E16.5 rat brain via IUE and were analysed at E19.5. As shown in Supplementary Fig. 9, the GFP levels in the cells expressing pCMS-eGFP were similar in the VZ and SVZ (Supplementary Fig. 9a). It was lower in the IZ, probably due to the dilution of plasmid during cortical development. However, the GFP levels in the cells expressing pCMS-eGFP-LBAL were downregulated significantly in both SVZ and IZ, reminiscent of the endogenous ATN1 levels (Fig. 4a and Supplementary Fig. 9b), suggesting the role of LBAL in the regulating ATN1 expression *in vivo*. Interestingly, when LSD1 was knocked down, the difference of GFP levels in the VZ and SVZ was compromised (Supplementary Fig. 9c), similar to the compromised expression of endogenous ATN1 in the VZ and SVZ of *LSD1* cKO mouse cortex (Supplementary Fig. 6c,d). These indicate that LSD1 regulates *ATN1* expression through the control of the histone methylation status at LBAL.

### ATN1 knockdown in NPCs leads to premature differentiation

**Figure 5:**
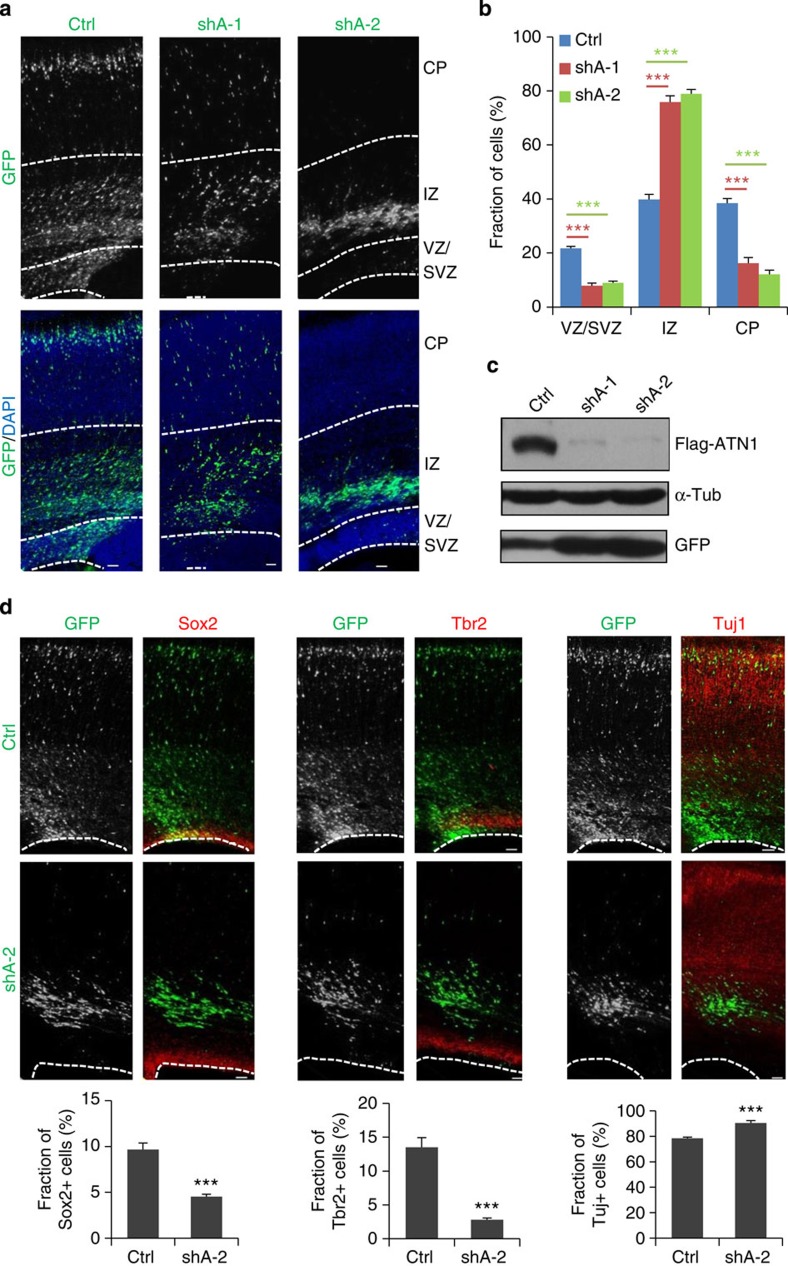
ATN1 knockdown phenocopies the depletion of LSD1. (**a**,**b**) ATN1 knockdown was performed as for LSD1 (Fig. 1d). Data represent mean±s.e.m. ***P<0.001 (Ctrl, *n*=21/7; shA-1, *n*=12/4; shA-2, *n*=27/9; one-way analysis of variance). (**c**) ATN1 can be efficiently knocked down by ATN1 shRNAs (shA). Flag-ATN1 was co-transfected with shCtrl or shAs into HEK293 cells; 24 h later, cell lysates were analysed by immunoblotting with anti-ATN1 antibody, with α-tubulin serving as loading control and GFP as transfection efficiency control. (**d**) ATN1 knockdown leads to premature differentiation of NPC. Data represent mean±s.e.m. ***P<0.001. Sox2 (Ctrl, *n*=5/5; shA-2, *n*=5/5); Tbr2 (Ctrl, *n*=4/4; shA-2, *n*=6/6); Tuj1 (Ctrl, *n*=5/5; shA-2, *n*=7/7); Student’s *t*-test. *n*, number of sections from different brains. Scale bar, 50 μm.

Given that LSD1 regulates *ATN1* expression, which is downregulated during NPC differentiation, the effects of ATN1 depletion on progenitor maintenance was examined. Two shRNA/eGFP constructs which were highly efficient at suppressing exogenous and endogenous *ATN1* expression (Fig. 5c and Supplementary Fig. 10a), were electroporated into E16.5 embryos and examined 4 days later. Their effects were similar to those observed on LSD1 knockdown; that is, fewer transfected cells were observed in the proliferative zones compared with controls, and cells failed to reach the CP (Fig. 5a,b). Co-transfection of a shRNA-resistant human ATN1 construct rescued the phenotype (Supplementary Fig. 11), although a slight delay in migration was still observed. In addition, no apparent increase of cell death was detected in ATN1 knockdown cells (Supplementary Fig. 10b).

The transfected cells were examined for cell fate marker expression to determine whether the loss of cells in the VZ and SVZ was due to premature differentiation of NPCs. The fraction of ATN1-deficient cells expressing the progenitor markers Sox2 and Tbr2 was dramatically lower than for control-transfected cells, while the fraction of cells expressing the neuronal protein Tuj1 was significantly higher (Fig. 5d). These results indicate that ATN1 knockdown leads to the depletion of the NPC population as a result of premature differentiation.

### LSD1 promotes NPC maintenance via regulation of *ATN1*

**Figure 6:**
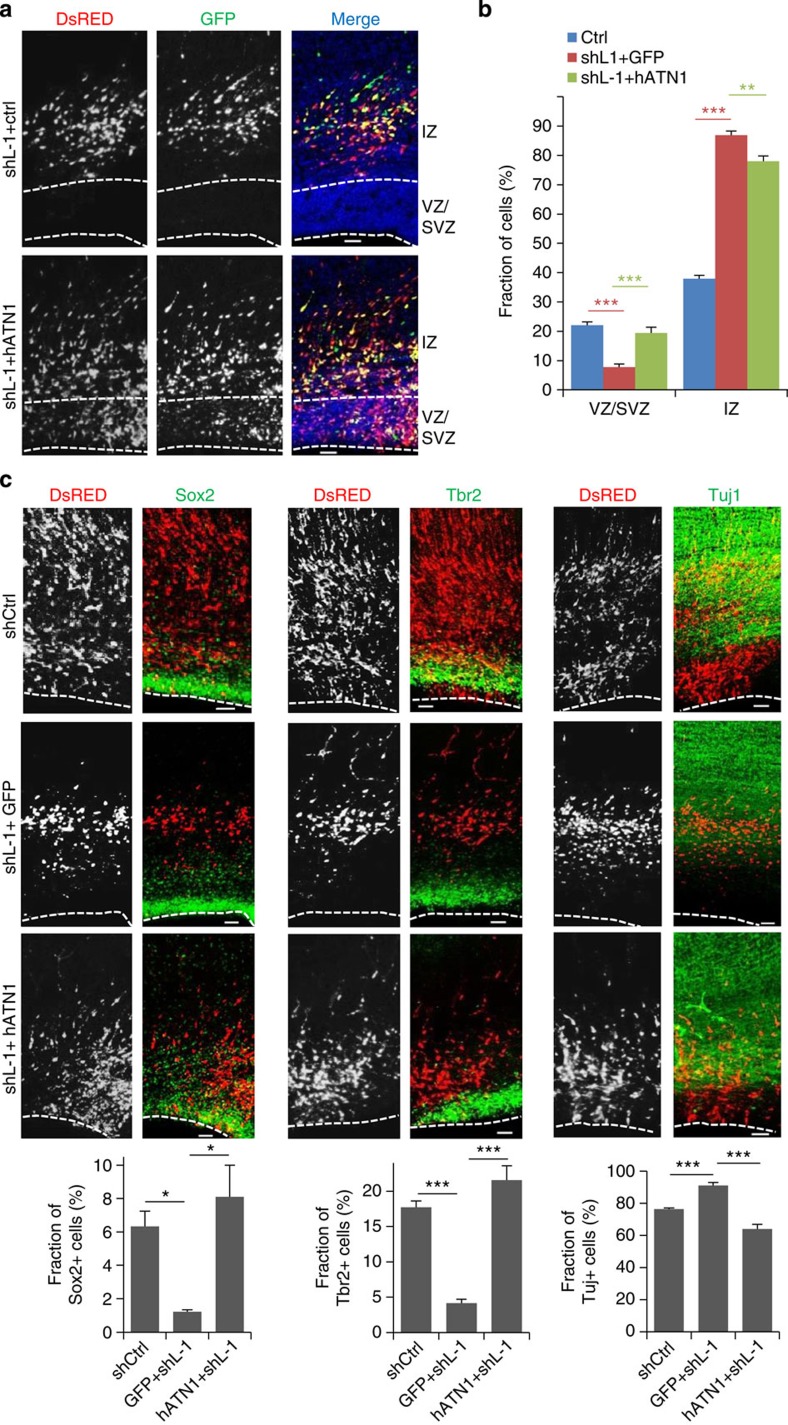
LSD1 maintains NPCs in a progenitor state via regulation of ATN1. (**a**) ATN1 overexpression rescues shLSD1-induced NPC depletion in the VZ and SVZ. Coronal sections of rat brains electroporated with shL-1 along with pCAGIG or pCAGIG-hATN1 at E16.5 and examined at E20.5. Scale bar, 50 μm. (**b**) Quantification of cell distribution in **a**. Data are means±s.e.m. **P<0.01, ***P<0.001 (Ctrl, *n*=26/8; shL-1+pCAGIG, *n*=8/3; shL-1+ pCAGIG-hATN1, *n*=10/3; one-way analysis of variance (ANOVA)). (**c**) Rescue experiments were done as in **a** and analysed as in (Fig. 5d). Data are means±s.e.m. *P<0.05; ***P<0.001. Ctrl (Sox2: *n*=7/5; Tbr2: *n*=5/5; Tuj1: *n*=7/5); shL-1+pCAGIG (Sox2: *n*=3/3; Tbr2: *n*=3/3; Tuj1: *n*=3/3); shL-1+ pCAGIG-hATN1 (Sox2: *n*=3/3; Tbr2: *n*=3/3; Tuj1: *n*=3/3). one-way ANOVA. *n*, number of brain slices from different brains (**b**,**c**). Scale bar, 50 μm.

**Figure 7:**
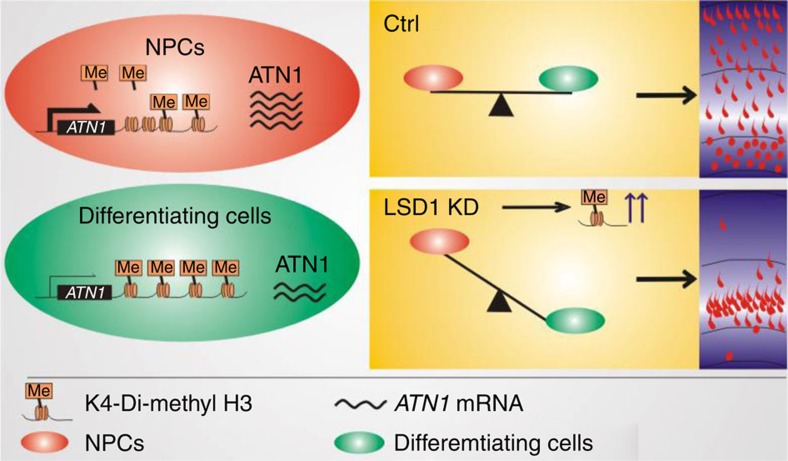
Model of NPC maintenance by epigenetic regulation of ATN1 by LSD1. In cycling NPCs, LSD1 maintains a low level of H3K4 dimethylation at the LBAL site of ATN1, resulting in a high level of ATN1 expression. In differentiating NPCs, H3K4 dimethylation at LBAL is increased, probably due to antagonistic methyltransferase activity, leading to decreased ATN1 expression and neuronal differentiation. When LSD1 demethylase activity is lost due to knockdown, knockout or tranylcypromine treatment, NPCs prematurely differentiate into neurons.

Given that depletion of ATN1 and LSD1 both induce premature differentiation, we went on to investigate whether the maintenance of NPCs controlled by LSD1 depends on the regulation of ATN1. E16.5 embryos were simultaneously electroporated with LSD1 shRNA and full-length ATN1 or control vector. The overexpression of *ATN1* rescued the depletion of NPCs in the VZ and SVZ caused by LSD1 knockdown (Fig. 6a,b). More importantly, expression of ATN1 rescued the LSD1 knockdown-induced reduction of Sox2^+^ and Tbr2^+^ cells, and increase of Tuj1^+^ cells (Fig. 6c), indicating that the loss of LSD1 in NPCs induces their premature differentiation into neurons through the deregulation of *ATN1* expression. Thus, we would like to propose that the demethylation of LBAL site downstream of *ATN1* locus by LSD1 maintains the pool of cortical progenitors through the induction of *ATN1* expression. Inhibition, knockdown or knockout of LSD1, as well as ATN1 depletion, causes NPCs to differentiate prematurely (Fig. 7).

## Discussion

Histone modification is a critical mechanism of gene regulation during development. Here we provide evidence that the demethylase LSD1 and its target gene *ATN1* are responsible for NPC maintenance during cortical development *in vivo*, providing another example of how specific epigenetic marks can direct cell fate choices. Premature NPC differentiation was associated with increased H3K4 dimethylation at LBAL, the LSD1-binding site of *ATN1* and lower levels of *ATN1* expression (Figs 2 and 3). Conversely, NPCs expressed *ATN1* at high levels, but had relatively low levels of dimethyl H3K4 (Fig. 4). These changes in methylation status are probably achieved through the antagonistic activities of a methyltransferase and LSD1. A reduction in demethylase activity by LSD1 knockdown, knockout or by treatment with a LSD1 inhibitor^18^, would lead to increased methylation at LBAL and downregulation of *ATN1* expression, resulting in NPC differentiation (model in Fig. 7).

A candidate methyltransferase in this model is the SET1/MLL histone methyltransferase complex. This is supported by two lines of evidence (Fig. 3a and Supplementary Fig. 8). First, depletion of RbBP5 and Dpy30, two subunits of the complex, produces an effect opposite to LSD1 knockdown; namely, a defect in *ATN1* repression during differentiation and thus the restriction of stem cell differentiation potential along the neural lineage^29^. Second, both Dpy30 and LSD1 bind to the LBAL locus^29^. Future studies can examine whether inhibiting the activity of the SET1/MLL complex can alter methylation at LBAL in cortical progenitors and induce an expansion of the progenitor pool.

Although we cannot exclude the possibility that LSD1 regulates *ATN1* expression levels through an indirect effect of blocking global histone demethylation, it is very likely to be that LSD1 regulates *ATN1* expression via controlling the histone methylation levels at LBAL. First, the expression of reporter gene with a downstream LBAL site can be suppressed by tranylcypromine treatment, similar to that of endogenous ATN1 (Fig. 3 and Supplementary Fig. 7). Second, reporter gene expression in cells transfected with pCMS-eGFP-LBAL is significantly lower in the SVZ than that in the VZ and this is compromised by LSD1 knockdown (Supplementary Fig. 9). Thus, it is reasonable to deduce that the downregulation of LSD1 level during NPC differentiation in the SVZ (Fig. 1a)^18^ leads to increased histone methylation levels at LBAL, and thus the corresponding decreased ATN1 expression in the SVZ as that of LSD1 (Figs 3, 4).

LSD1 has been shown to remove both the mono- or dimethylation of H3K4 and H3K9 (refs 13, 30). Although we are not certain that downregulation of *ATN1* expression by LSD1 inhibition is caused by increased monomethyl H3K4 or dimethyl H3K9 at the LBAL site, this kind of regulation is more likely to be mediated by dimethyl H3K4 for two reasons: One is that the H3K9 methylation levels at LBAL site in NPCs are much lower compared with that of H3K4 methylation (Figs 3d, 4f); The other reason is that when NPCs undergo differentiation, it is changes of dimethyl H3K4, but not monomethyl H3K4 levels, that are accompanied by the alternation of *ATN1* expression (Fig. 4f).

Although H3K4 trimethylation in promoter region has been observed to repress transcription^31^, tri- and dimethylated H3K4 are predominantly associated with the activation of gene expression^6^. This maybe the reason why LSD1 is mostly reported as a transcriptional repressor^16^,^32^. In the present study, inhibition of LSD1 activity resulted in enhanced H3K4 dimethylation at LBAL in NPCs, accompanied by the downregulation of *ATN1* expression. It is interesting to speculate that this contradictory effect is due to the location of LBAL downstream of the *ATN1* coding region, instead of within the promoter as is typically the case. In this instance, a high dimethyl H3K4 level at LBAL might produce a conformational change in the chromatin that would favour transcriptional repression, perhaps by physically obstructing the access of the transcriptional complex to the promoter. Although LSD1 activates androgen receptor transcription by removal of mono- and dimethyl H3K9 (instead of H3K4) histone marks^14^,^30^, to the best of our knowledge our study is likely to be the first report that LSD1-involved H3K4 methylation is required for gene activation as well as NPC maintenance.

Even though the present results indicate that *ATN1* is probably a direct target of LSD1 in the regulation of NPC maintenance, there are some aspects of the mechanism that remain unclear. For instance, ATN1 has been reported to interact with and modulate the repressor activity of TLX^32^, while loss of TLX function also induces premature differentiation of NPCs^33^, raising the possibility that ATN1 exerts its effect on progenitor fate through TLX. LSD1 and TLX have also been shown to directly interact and coordinately repress target gene expression^16^,^32^. Therefore, LSD1 could regulate ATN1 directly, or else indirectly through TLX. Alternatively, LSD1, ATN1 and TLX could interact within a single complex. Reduced levels of ATN1 could disrupt the repressor complex that normally suppresses differentiation and maintains progenitors in a state of self-renewal, resulting in the derepression of differentiation-promoting genes.

In addition to the epigenetic regulation of *ATN1* by LSD1 during embryonic brain development, *ATN1* expression in the adult brain would be investigated in the future to determine whether it can be suppressed by the LSD1 inhibitor tranylcypromine, which is currently prescribed for the treatment of mood and anxiety disorders in the clinic. If that is the case, given that DRPLA is caused by the aggregation of ATN1, tranylcypromine could be tried for the prevention and alleviation of some DRPLA symptoms.

## Methods

### Plasmids

*LSD1*-specific shRNAs, shL-1 and shL-2 were constructed into RetroQ-DsRed-shRNA. Target sequences (for rat *LSD1*) of shControl: 5′- GTGCGTTGCTAGTACCAAC -3′; shL-1: 5′- GGCGAAGGTAGAATACAGA -3′; shL-2: 5′- GGATGGATGTCACACTTCT -3′. *ATN1*-specific shRNAs, shA-1 and shA-2, were cloned into pLL3.7-GFP. Target sequences (for rat *ATN1*) of shcontrol: 5′- TCATGCCTCTATCTACGTC -3′; shA-1: 5′- CGAGAACGAAGTTCTTCGT -3′; shA-2: 5′- GGGCCTTCAAGTTTGTCAT -3′. Flag-tagged human *ATN1* were from Dr Chih-Cheng Tsai^34^, and were recloned into pCAGIG. Rat *ATN1* was amplified by PCR from E16.5 rat brain complementary DNA and cloned into pCMV-Tag2b. Flag-tagged human *LSD1* was from Dr Zengqiang Yuan. Human *LSD1* was synonymously mutated into a form refractory to shL-1 and cloned into pCAGIG. (from 5′- ggcgaaggtagagtacaga -3′ to 5′- ggcCaaAgtGgagtacaga -3′). Rat *LSD1 ATN1* was amplified by PCR from E16.5 rat brain cDNA and cloned into peGFP-N1. The LSD1-binding site around *ATN1* locus was cloned from rat genome and inserted into the EcoR1/Sal1 site of pCMS-eGFP. The primers used for cloning are listed in Supplementary Table 2.

### Cell culture and transfection

HEK293 cells and N2a cells were cultured in DMEM (neuronbc) with 10% fetal bovine serum (Excel) and 1% penicillin/streptomycin (P/S, Gibco), and plasmids were transfected into 293 cells with VigoFect (Vigorous) and into N2a cells with Lipo2000 (Invitrogen).

E16.5 rat primary NPCs were cultured in dishes pre-coated with poly-D-lysine and in the DMEM medium with 1% B27 supplement (Gibco), bFGF (20 ng ml^−1^, R&D), EGF (20 ng ml^−1^, R&D), 1% P/S or DMEM with 1% N2 supplement (Gibco), bFGF and 1% P/S. Neurospheres were cultured as described^35^. Briefly, E16.5 rat brains were dissected and digested with 0.25% (W/V) Trypsin-EDTA for 20 min and then disassociated and cultured in DMEM with 1% B27 supplement, bFGF, EGF and 1% P/S.

### Western blotting and immunostaining

Western blotting and immunostaining were performed as described^36^,^37^. Briefly, the protein samples were separated by SDS–PAGE and transferred to nitrocellulose membranes. The nitrocellulose membrane was incubated with the primary antibody at 4 °C overnight and then with the horseradish peroxidase-conjugated secondary antibody for 1 h. Pierce ECL (enhanced chemiluminescence) western blotting substrate was used to visualizing the protein bands. The antibodies used for western blotting were as follows: GFP (Abcam, ab290, 1:2,000), α-tubulin (CST, 3873s, 1:2,000), GAPDH (CST, 2118s, 1:2,000), LSD1 (CST, 2139s, 1:1,000), ATN1 (Sigma, HPA031619, 1:1,000), Flag (MBL, M185, 1:2,000) and DsRed (MBL, PM005, 1:1,000). For immunostaining experiment, cultured cells or brain slice were fixed with 4% paraformaldehyde. After incubation with the primary antibody at 4 °C overnight and then with fluorescent-conjugated secondary antibody for 1 h, images were acquired with Zeiss LSM700. The antibodies used for immunostaining were as follows: LSD1 (CST, 2184s, 1:400), ATN1 (Sigma, HPA031619, 1:400), Sox2 (Abcam, ab97959, 1:1,000), Tbr2 (Millipore, ab2283, 1:1,000), β-III Tubulin (Abcam, ab7751, 1:1,000), Nestin (Abcam, ab6142, 1:1,000), GFP (Abcam, ab13970, 1:1,000), DsRed (MBL, PM005, 1:1,000) and activated-caspase3 (Abcam, ab13847, 1:1,000). Images of uncropped western blottings are shown in Supplementary Fig. 12.

### Animals and *in utero* electroporation

*LSD1*^*flox/flox*^ mice were kindly provided by Dr M Rosenfeld’s group and Nestin-*Cre* mice were purchased from Model Animal Research Center of Nanjing University. Pregnant Sprague–Dawley rats and adult ICR female mice were provided by the animal centre of the Institute of Genetics and Developmental Biology (IGDB), Chinese Academy of Sciences, and the experimental procedures were performed according to protocols approved by the Institutional Animal Care and Use Committee at IGDB.

IUE was performed as we described previously^38^. Briefly, the plasmids were injected into the lateral ventricle of E15.5 or E16.5 rat brains, which were given an electrical stimulation (50 V for 50 ms with a 950 ms interval) for 5 times. Three or 4 days later, the rats were killed and embryonic brains were fixed in 4% paraformaldehyde and dehydrated in 30% sucrose. The brains were frozen-sectioned into 50-μm slices with Leica CM1950.

### Imaging and statistical analysis

Confocal images were achieved through Zeiss LSM700 and analysed with Photoshop, ImageJ or Imaris. The data were analysed with *t*-test, paired *t*-test or one-way analysis of variance and the significant difference was shown as *P<0.05, **P<0.01 and ***P<0.001. The fraction of Sox2+ cells (and Tbr2+ and Tuj1+ cells) was calculated by dividing number of all cells transfected with shRNAs by number of Sox2+ (and Tbr2+ and Tuj1+ cells) cells transfected with shRNAs across the cortex. The number of brain slices analysed was displayed in ‘*n*=*a*/*b*’, where ‘*a*’ is the total number of brain slices analysed and ‘*b*’ is the number of different mouse or rat brains.

### RNA extraction and real-time PCR

RNA extraction with TRIZOL was performed as described^39^. Cultured cells or brain tissues were dissolved in 1 ml TRIZOL and 200 μl chloroform was then added. After precipitation with isopropanol and washing with 70% (V/V) ethanol, the collected mRNA was reversely transcribed into cDNA with reverse transcriptase (Promega), and real-time PCR was performed with Bio-Rad C1000 Thermal Cycler. The primers used are listed in Supplementary Table 2.

### ChIP and ChIP-seq

ChIP with LSD1 antibody (CST, 2184s) and Di-Methyl-Histone H3 (K4) antibody (CST, 9726s) was performed with SimpleChIP Kit (CST) according to manufacturer’s protocol. Purified DNA from ChIP samples with LSD1 and IgG antibodies from E16 rat brains were then sent to Beijing Genomics Institute (BGI, China) for sequencing and data analyses. Our ChIP-Seq data has been deposited in the GEO database under the accession number of GSE62770.

## Author contributions

F.Z. designed and performed most of the experiments. D.X. performed some of the IUE and immunostaining analysis. L.Y. helped with some western blot analysis. Y.S. and Z.X. were involved in study design. All authors discussed the results and commented on the manuscript.

## Additional information

**How to cite this article**: Zhang, F. *et al*. Epigenetic regulation of *Atrophin1* by lysine-specific demethylase 1 is required for cortical progenitor maintenance. *Nat. Commun.* 5:5815 doi: 10.1038/ncomms6815 (2014).

## Supplementary Material

Supplementary Information Supplementary Figures 1-12 and Supplementary Tables 1-2
