# Supplementary material for: Epigenetic regulation of Atrophin1 by lysine-specific demethylase 1 is required for cortical progenitor maintenance
Source: Nat Commun. 2014 Dec 18;5:5815. doi: 10.1038/ncomms6815 (PMC4284801; doi:10.1038/ncomms6815)
Supplement: Supplementary Information — Supplementary Figures 1-12 and Supplementary Tables 1-2 [file ncomms6815-s1.pdf]

Supplementary Figure 1.

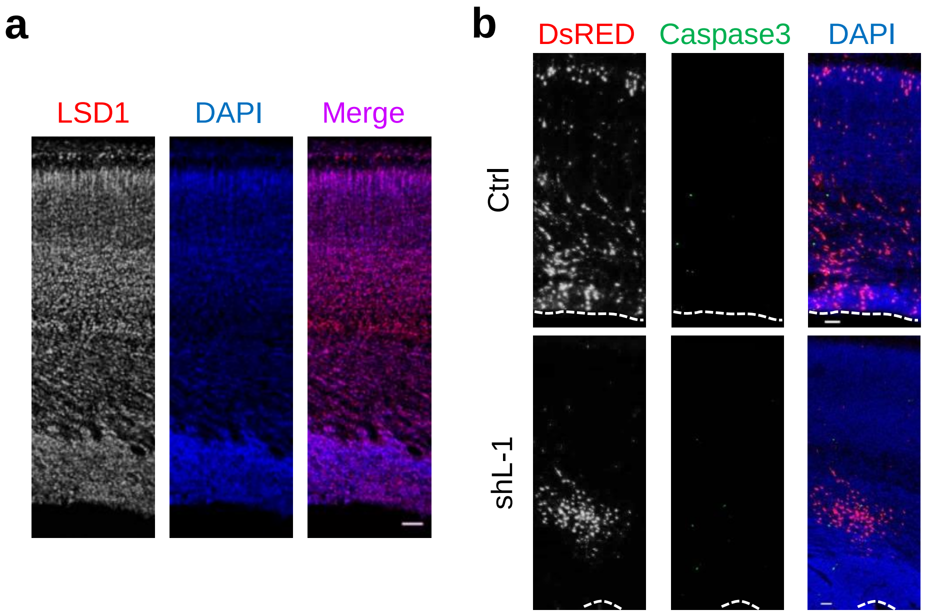

Supplementary Fig. 1 related to Fig. 1. **LSD1 is expressed during brain development and LSD1 knockdown does not induce obvious apoptosis. (a)** Immunostaining of coronal rat cortical sections at E20.5 with LSD1 antibody. Nuclei were labeled with DAPI. Scale bars, 50μm. **(b)** Brain slices (E20.5) electroporated with control or shL-1 at E16.5 were immunostained with activated-caspase3 antibody to label cells undergoing apoptosis. Scale bar = 50 μm.

Supplementary Figure 2.

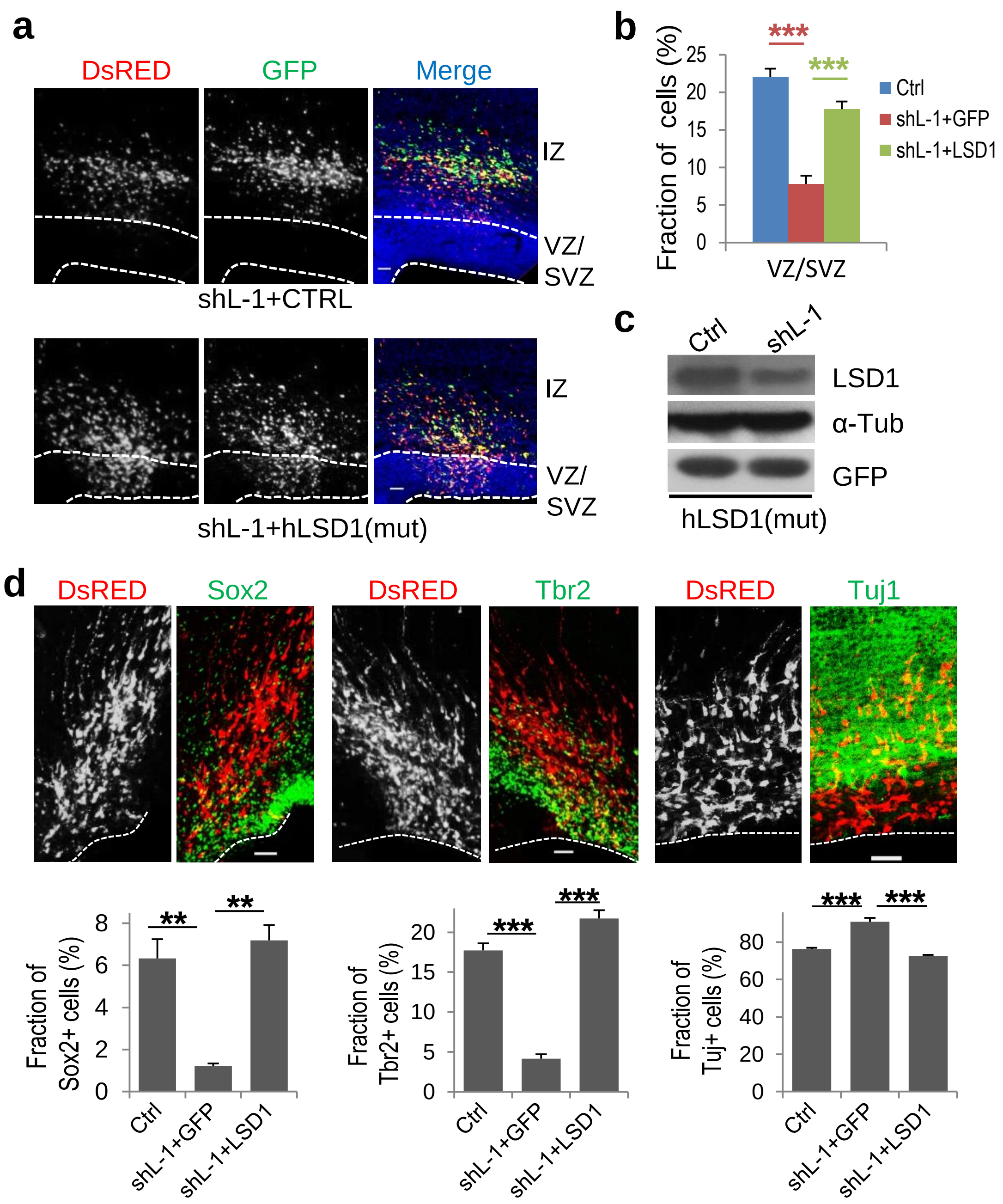

Supplementary Fig. 2 related to Fig. 1&2. **LSD1 knockdown induced NPC depletion can be rescued by synonymously mutated human LSD1. (a)** Coronal sections of rat brains electroporated with LSD1 shRNA (shL-1) together with pCAGIG-GFP or pCAGIG-hLSD1(mut) at E16.5 and inspected at E20.5. Scale bars, 50μm. **(b)** Quantification of cell distribution in (a). Data are means±SEM. ***: P<0.001. One-way ANOVA. shCtrl: n=26/8; shL-1+GFP: n=8/3; shL-1+hLSD1: n=10/3. n: number of brain slices from different brains. **(c)** PCAGIG-hLSD1(mut) (synonymously mutated human LSD1) were cotransfected with control shRNA (shCtrl) or shL-1 into HEK293 cells and analyzed as in Fig. 1b,c. **(d)** Rescue experiments were done as in (a) and analyzed as in (Fig.2a). Data are means±SEM. **P<0.01; ***P<0.001. Ctrl (Sox2: n=7/5; Tbr2: n=5/5; Tuj1: n=7/5); shL-1+pCAGIG (Sox2: n=3/3; Tbr2: n=3/3; Tuj1: n=3/3); shL-1+pCAGIG-mut-hLSD1 (Sox2: n=4/3; Tbr2: n=4/3; Tuj1: n=4/3). one-way ANOVA. n: number of brain slices from different brains. Scale bars, 50 μm..

Supplementary Figure 3.

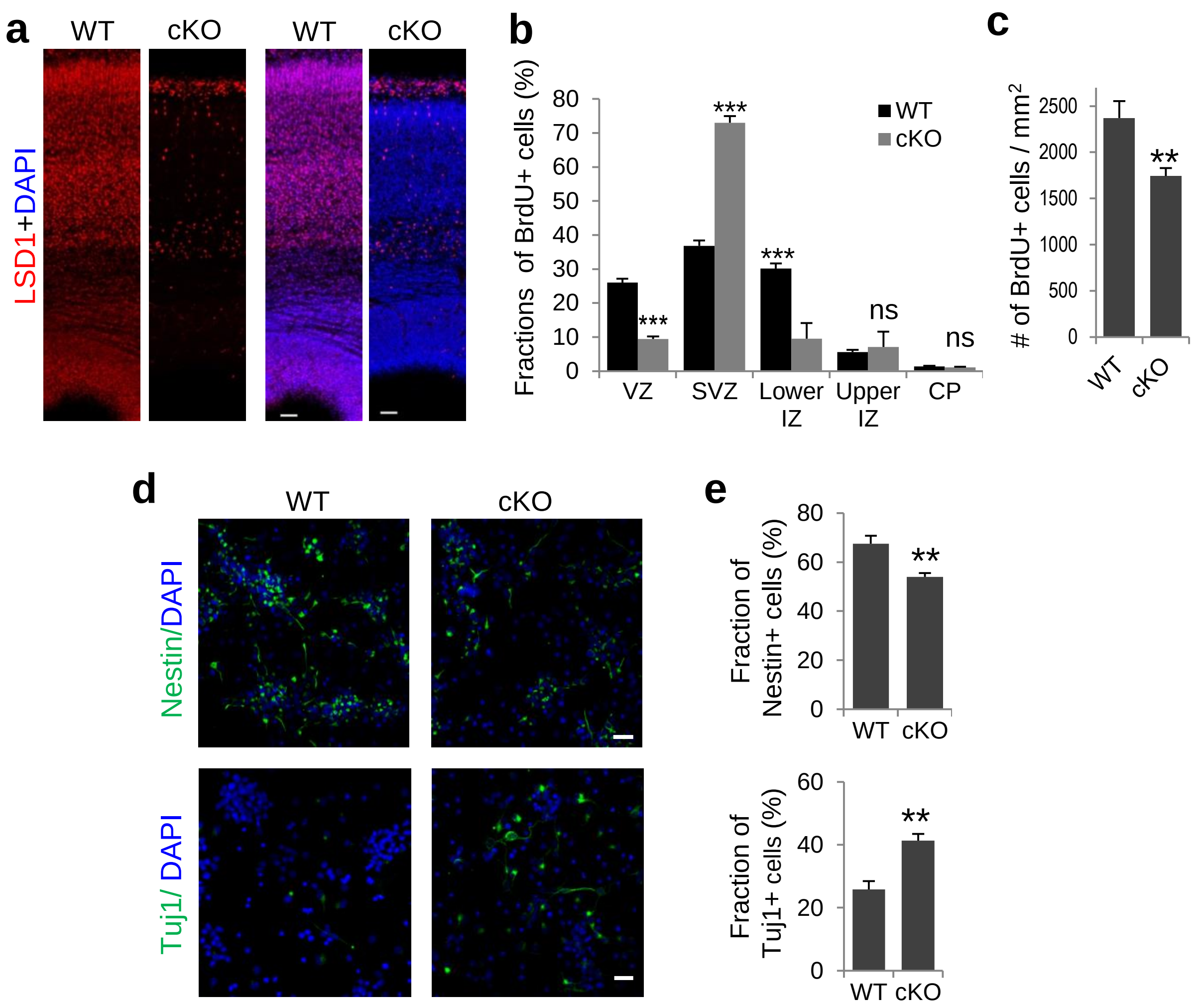

Supplementary Fig. 3 related to Fig. 2. **Knockout of LSD1 leads to enhanced differentiation of NPC.**

(a) Brain-specific *LSD1* cKO leads to thinner cortex. Immunostaining of coronal cortical sections from P0 wide-type or *LSD1* cKO mice with LSD1 antibody. Nuclei were labeled with DAPI. Scale bar = 50 μm. **(b, c)** Wide-type or *LSD1* cKO mice were treated and analyzed as in Fig. 2c,d. (b) Quantification of BrdU+ cell distribution across the coronal cortex. (c) Quantification of the number of BrdU+ cells in the cortex (per mm^2). Data represent means ± SEM. ***$P < 0.001$, **$P<0.01$, ns, not significant. (WT: n=9/3; cKO: n=9/3; Student's t- test). n, number of slices from different brains. **(d,e)** NPC from E14.5 mouse cortices were cultured for 48 h, stained for Nestin and Tuj1 and quantified. Data represent means ± SEM. **P (Nestin)=0.008; **P (Tuj1)=0.004 (t-test, >1500 cells were counted for each group from four experiments). Nuclei were labeled with DAPI. Scale bar = 50 μm.

Supplementary Figure 4.

```
>chr4:161114749-161117223
CGGAGGCAGAGGTAGATGGAGAATCTCTGAGTTCGAGACCTGCGTGGTCTACAGAACGAGTTCCAAGATAGCCAGGGCTTTGG
GCTTAAGAGCCAGAACTTTTTTGTTGTTGTTTGTTTTTCAAGACAGGGTTTCTCTGTGTAGCCTGACTGTACCGGAACGCCTCACC
CTACAGACCGAGGCTGGCTTCGAACTCAGAGATCTGCCTGCTTCTGCCTCCTAAATGTCGGGATGAAAGGCGCATGACAGCACT
GCCTGGTTTCAAGAGTCAGAACATTTTAGGCCGGGCTTAAGTATAGTCCCAGAGCTGTGAGTGGCAATGTTTGCTGTGACTGCC
GCCCTGCGGCGAGAAAGAAATCGACCCAGGAGCCATCATGCGCCCCACCCCCAGTCCATTCCATCGCCCTTCCCTGTAGGACC
CACCTTCCCCGTCGCAGCTGAAGCCATAGGCTTTAATAACCTCCTGCTGGATCTGTGTGGCTACAGGCAGCACGAATTGCAGCA
TCTTGCCCATATCGTTGCACGCATTGTCTCTAGCCTCGTCCATACGCACGGCGTTCTCTGGGGCCGAGAACGCTTGGATCACCT
CCGCCAAGACCACTGCGAGACCGAAGCATCCCAGAGAGGTCCGCGGAGGACGGCGGGCGAGCCCAGAGAAGGGGAGAGCCA
GGGAATTAGTGGACCGAGGCCTGCTTATCTGGAGGATAAGCTCGTCCTCCCAACCCTTCGCCCTAATGGGCAAGGGCCCCACT
TGGAGAAGGAACGAATAGTACTGCGCCCTCCCCCCAGAAGACGGCCCACGCCCAGACTAGGCTCCTCACCCTTGGCCTGCTCT
GCGCTCAGAGCCGCAGGCTGAGCCGAGGCGGACGCCATAGGACGCCACTCGGTGCTTGAATAGTGTGAACACTGAGATCCGG
AGGAGCCTGCAGCCAGCCGCCTCCCCCACGGCTGCGGACCTGTGGAGGCAGAAAGGAAGCTGTATTGCAGGAGGCGGAAGTT
CCCGAGCGTGAATCTAGCACACGCGTACTTCCTAGGAAACTAGAGTGGGCCGAAAGCCCCCAGCCCCATAGCTCGGCGAGGAC
AGTAACTACTTGCTGATATTAGAAACCAGTCAGGTTGGGAGGGGTTTTCCGACCGAAGTCAGAAAACCTACTAGACAAATTCTAA
AAGAGCTGTAACACTTGGCGGAAGCTATGCAGTAGCGAGCCAGGGAAGGACGTCAACTCCACTTTTGGTGAGGGTGAGATCAG
GGTGCCATTTCCACATCCCTCCACCAATATATGAATCATAAAGCATACTTTCTTATTCAGTTCGGTAAATAATATTCTAAAAGTCTA
CTAAAACCAGTCATTTCTTGAGTTTGTGACCACTTAATAGGACTATGCAAATGACTCAATGCTGATTGGCTGAAAACAGCCAATCA
CAGCCCCTTCGTTGTTAAGAGGGAAAAAAAGAGCTTCTCAAGAATAAGAGCTAGGAGAGCAAAAGCCGCCGTGAACTGATCTTA
CGGTAGTGGTAGGCGAAATGGCTCGATGGCTTGTATTCTTTTTTTTTTTTTTTCTCTTTTCTCTTTTTTCGTTTTTCCGGAGCTGGG
GACCGAACCCAAGGCCTTGCGCTTGCTAGGCAAGCGCTCTACCACTGAGCTAAATCCCCAACCCCGATGGCTTGTATTCTTGTC
TTTACAAGTTTTTGTCACCCATCCTCTCCTCTCACACTACAAGGACTCTGGAACCTCAAGCCCTACCTGGCATGGTGCCACACTT
CTGTGATACCAGCACAAAAACTGAGAGGAAGTTCAAGGCTAGCTTGGACTCCATGGCCCTGCTTTGCTCAACCTACTTGGCAATT
CCTAACTTTCCTAATTCAAGCTACAGGGGACCCAACACCTTCTGGCCTCTTTTGAGGCCTTGCATTCCTAAGCAGAGACATGAAC
CCACACACCTAATTAGAAATAAATCTTTTAAAAATAAATCTTATTGGCCTCCTGGTTACACAATTTAACTGTTTCAAAATCATTTATA
AACTATTGCCACGGAAAGGGAGTCAGATCTCTGAAATTCTGTCTCTGGAGCACTCTGGTGGCAGGGATGTACTCCCAAGGTCTG
AGTAGGTAGATTTGGTTATGCTTACTGTGAACCTATTCTTGCTCTGAAAGGCCTCAAGGGCCTTAACTCCCCAATCCAAAGGTCT
AGGATGGGCGCCCCATGAGTAATTCTGAGATTCCAAGACCAAGAAGAGCCTTGTCAGGCTAAAGAGTGGCTCAGTGGTTAAGA
GCAGACTGCTCTTCCAGAGGTCCTGAGTTCAATTCCCAACAAACACATGGTGGCTCACAGCCATGTGGAATGGGATCCGATGCC
CTCTTCTGGTGTGCTTCATGCAAGTGTAAAATAAATGAATAAAAAATTTAAAAAAAAGAACCTTGTCCAACTCATCTGTTTCCATAA
GGAAACCCCATCCATCCCTCCACACTTGCCC
```

Supplementary Fig. 4. related to Fig. 3. **The sequence of rat LBAL site achieved by ChIP-seq.** The underlined letters are the parts used for the design of primers.

Supplementary Figure 5.

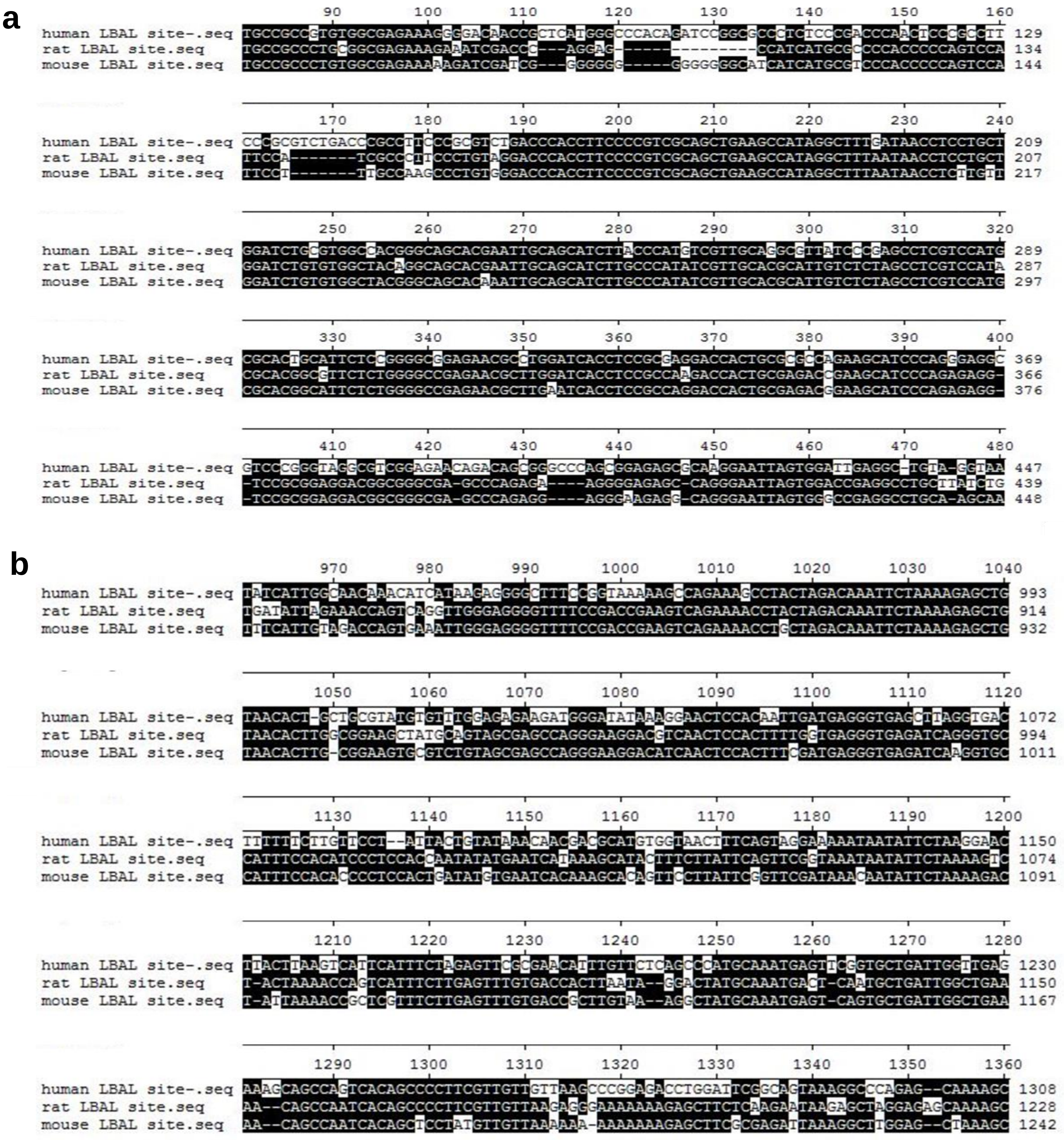

Supplementary Fig. 5. related to Fig. 3. **LBAL, LSD1 binding site at the ATN1 locus, is conserved in rodents and humans. (a, b)** Schematic illustration of sequence alignment of LBAL site from human, rat and mouse. Blast results showed that 85% nucleotides of one part of rat LBAL site are identical to those of mouse LBAL site (1108 out of 1306 nucleotides are the same). Two major parts of LBAL site (a and b) are homologues between human and rat. 244 out of 297 nucleotides in (a) and 227 out of 328 nucleotides in (b) are identical. The letters highlighted in black color showed that human, rat and mouse share the same nucleotides there.

Supplementary Figure 6.

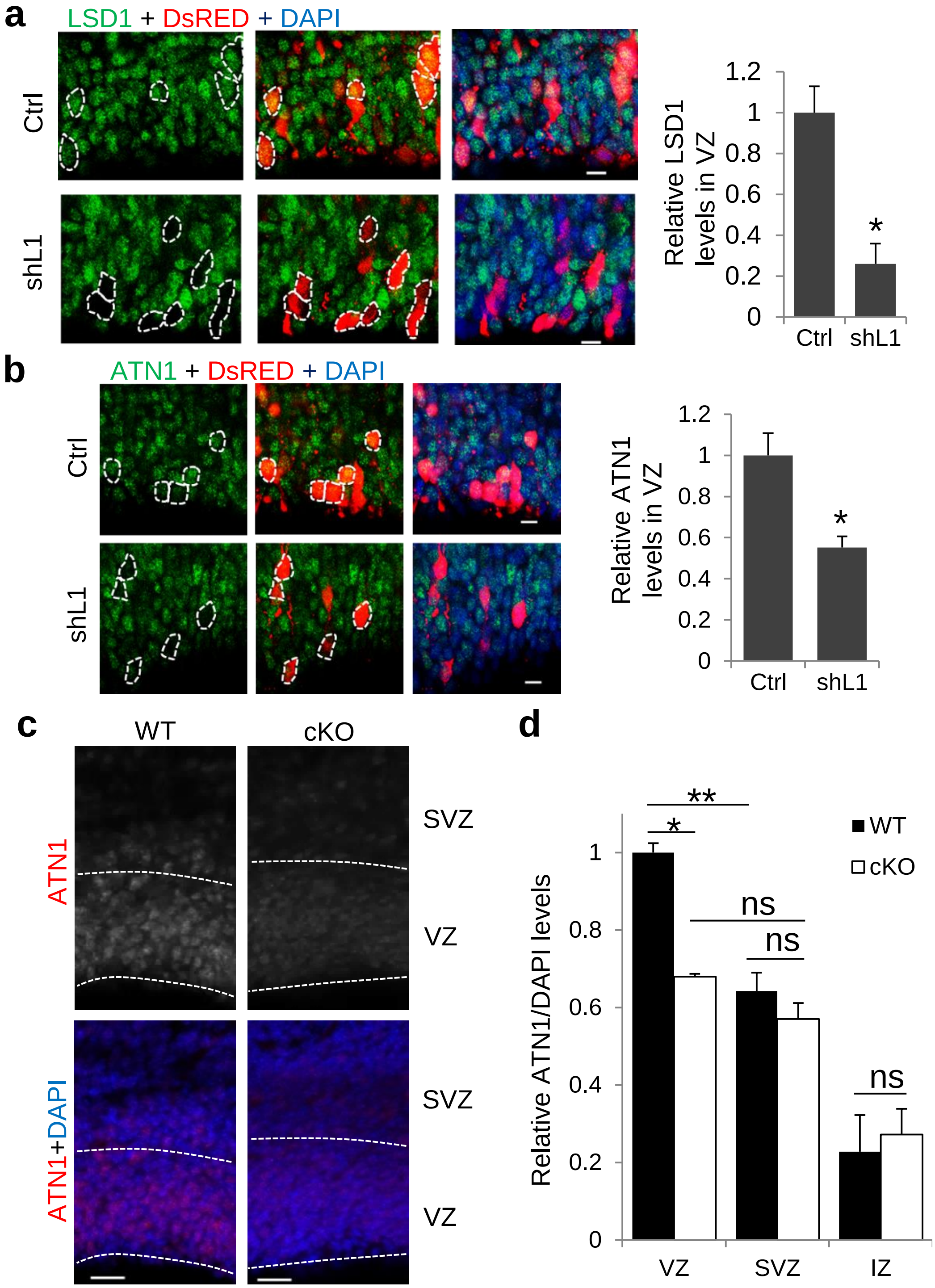

Supplementary Fig. 6 related to Fig. 3. **Knockout or knockdown of LSD1 leads to down-regulation of ATN1 level. (a,b)** Immunostaining of coronal cortical sections from E19.5 rat brain electroporated with Ctrl or shL1 at E16.5 with LSD1 (a) or ATN1(b) antibody. For each cortex, the fluorescence intensity of 5~10 DsRED+ cells in VZ was quantified and the average was used to represent as the levels of LSD1 or ATN1 of this cortex. Then the levels of LSD1 or ATN1 in shRNA group were normalized to that of shControl group. Nuclei were labeled with DAPI. *P=0.01 (LSD1), *P=0.02 (ATN1). LSD1 (Ctrl: n=3/3; shL1: n=3/3); ATN1 (Ctrl: n=3/3; shL1:n=3/3); Student's t-test. n: number of brain slices from different brains. Scale bar: 5μm. **(c,d)** Immunostaining of coronal cortical sections from wild-type or brain-specific *LSD1*-cKO mice at P0 with ATN1 antibody. Nuclei were labeled with DAPI. Data are means ± SEM. *P<0.05; **P<0.01; ns, not significant. (WT: n=6/3; cKO: n=6/3; one-way ANOVA). Scale bar: 20μm.

Supplementary Figure 7.

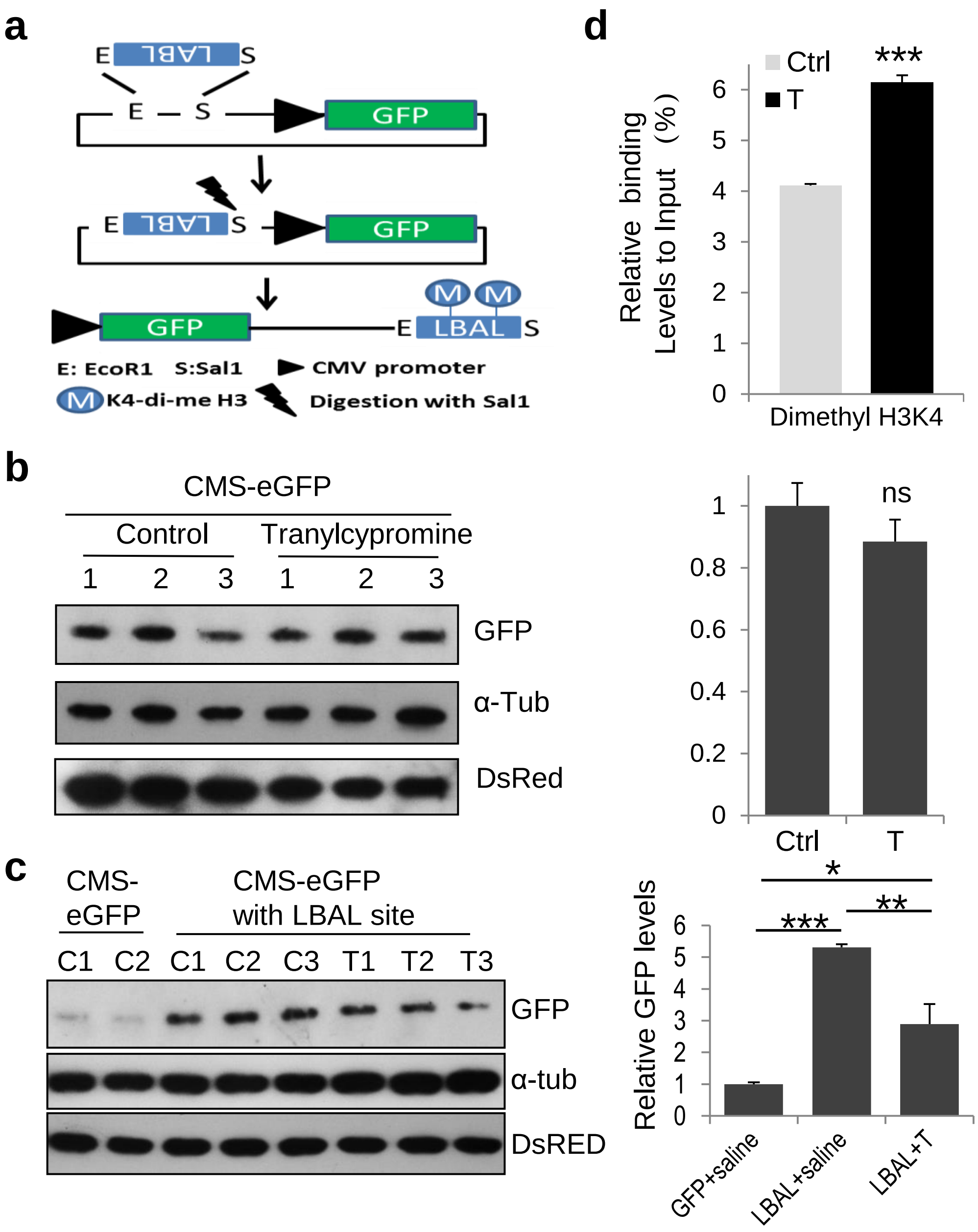

Supplementary Fig. 7. related to Fig. 3. **LBAL site plays an important role in gene expression.**

(a) Schematic illustration of pCMS-eGFP-LBAL reporter. LBAL site was inserted into pCMS-eGFP at EcoR1/Sal1 site. The construct was cut with Sal1 to generate pCMS-eGFP-LBAL. **(b,c)** Linerated pCMS-eGFP or pCMS-eGFP-LBAL were transfected into N2a cells together with RetroQ-DsRED. 4 h later, cells were separated into two equal parts. One part was treated with saline and the other part with Tranylcypromine (T, 10μM). 16 hrs later, cell lysates were collected and GFP levels were analyzed by western blotting, with α-Tub as the loading control and DsRed as transfection efficiency control. Right panel: Quantification of GFP expression levels with α-Tub as the loading control. Data was shown as means±SEM. *P<0.05, **P<0.01, ***P<0.001; (n=3; T-test). **(d)** N2a cells were treated as in (c). Histone modification levels at LBAL site in the linearized plasmids were analyzed as in Fig. 3d. Data was shown as means±SEM. ***P<0.001; ns, not significant. (n=3; t-test).

Supplementary Figure 8.

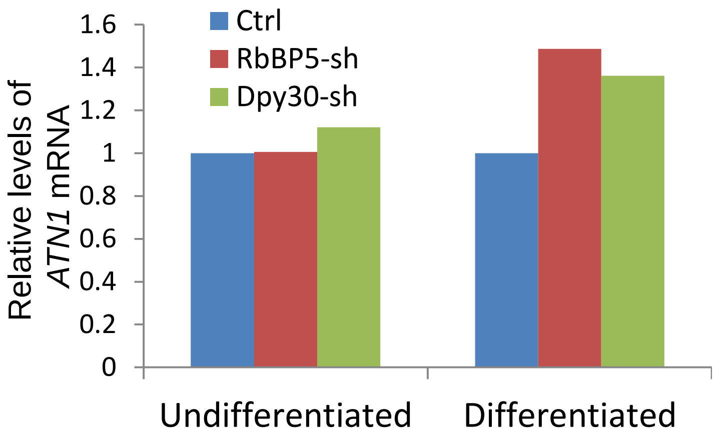

Supplementary Fig. 8. related to Fig. 3. **Depletion of RbBP5 or Dpy30 induces ATN1 expression.**

Depletion of RbBP5 or Dpy30 (subunits of histone methyltransferase SET1/MLL complexes) abort the ability to down-regulate ATN1 expression in embryonic stem cells (ESCs) undergoing differentiation (treatment with retinoic acid), while have little effect in undifferentiated ESCs. Quantification analysis of ATN1 levels from gene-chip results and deposited in the GEO database under GSE26136. (Jiang H, Shukla A, Wang X, Chen WY, Bernstein BE, Roeder RG (2011) Role for Dpy-30 in ES cell-fate specification by regulation of H3K4 methylation within bivalent domains. Cell 144: 513-525).

Supplementary Figure 9.

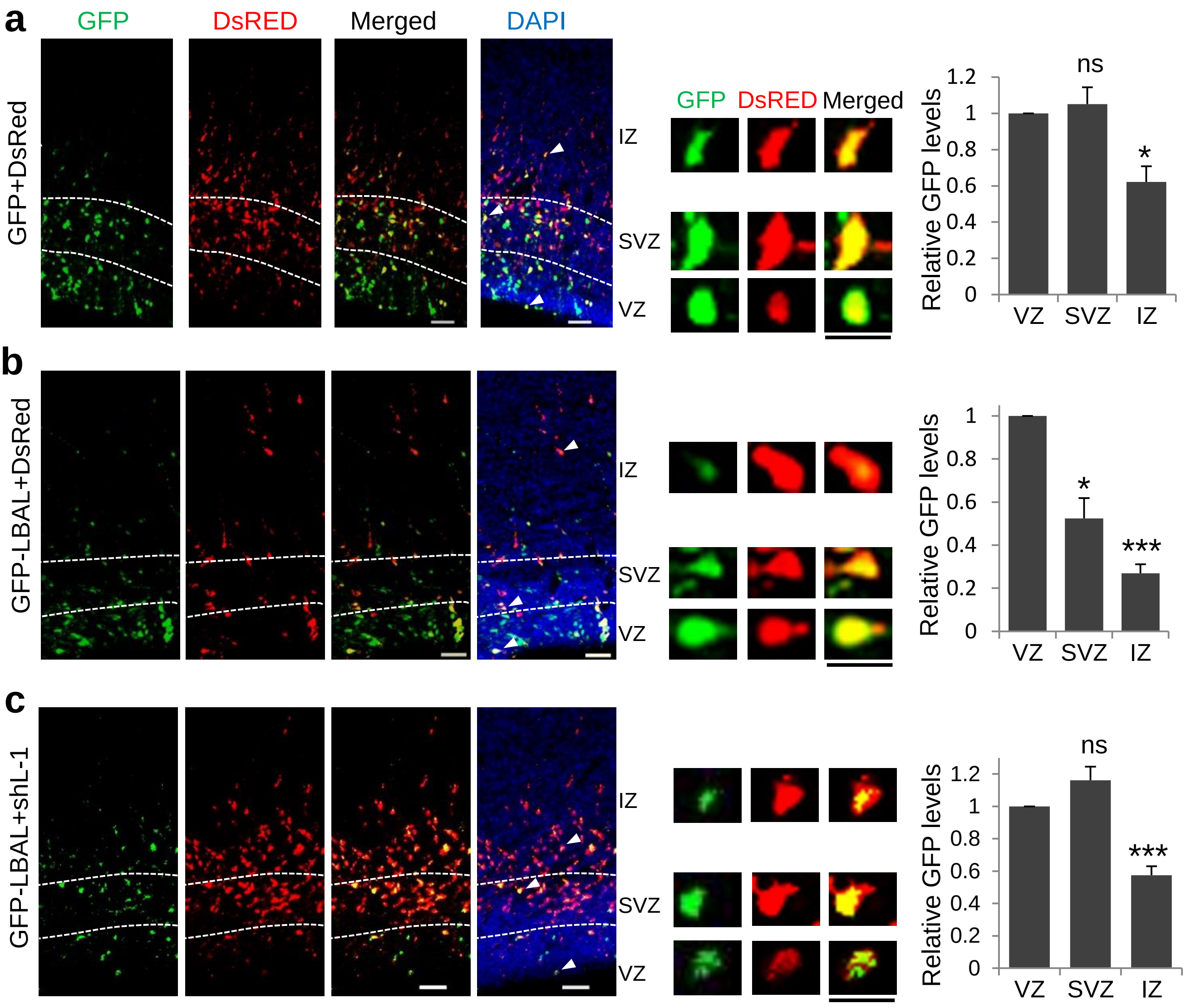

Supplementary Fig. 9. related to Fig. 4. **LBAL site plays a role in gene expression in the developing brain.**

(a-c) Coronal sections of rat brains electroporated *in utero* with linerated-pCMS-eGFP and DsRED (a), linerated-pCMS-eGFP-LBAL and DsRED (b) or linerated-pCMS-eGFP-LBAL and shL-1 (c) at E16.5 and examined at E19.5. Middle panels: enlarged image of representative cells (indicated by the white arrowhead in the left panel) from different layers (VZ, SVZ, IZ). Right panels: quantification of the intensity of eGFP in cells distributed in different layers (VZ, SVZ and IZ). Data represent means ± SEM. *P<0.05, ***P<0.001; ns, not significant. (pCMS-eGFP+DsRED: n=6/3; pCMS-eGFP-LBAL+DsRED: n=5/2; pCMS-eGFP-LBAL+shL-1: n=6/3; paired t-test). n: number of brain slices from different brains. Scale bar: 50μm (left panels); 20μm (right panels) .

Supplementary Figure 10.

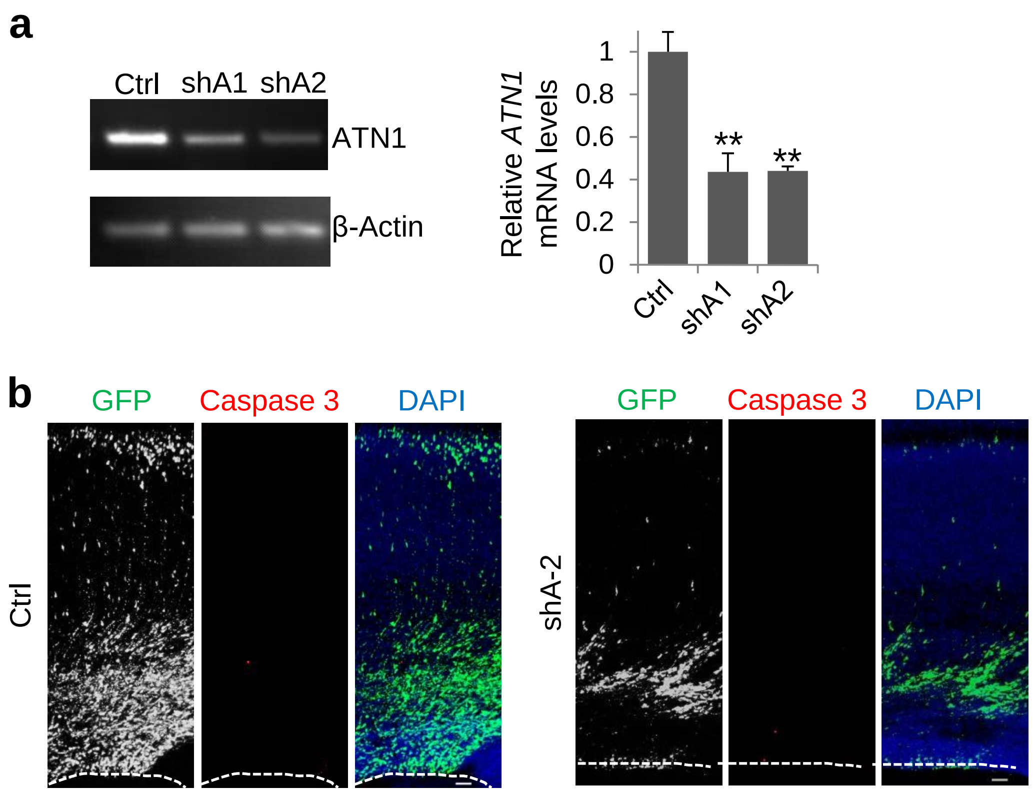

Supplementary Fig. 10. related to Fig. 5. **Endogenous ATN1 is efficiently knocked down by ATN1 shRNAs and ATN1 knockdown does not induce obvious apoptosis.**

(a) Rat brains were electroporated with Ctrl, shA-1 or shA-2 at E16.5 and GFP+ cells were collected by FACS at E19.5. *ATN1* mRNA levels in these cells were analyzed by qPCR. **P< 0.01, (n=3; one-way ANOVA). **(b)** Brain slices (E20.5) electroporated with control or shA-2 at E16.5 were immunostained with activated-caspase3 antibody to label cells undergoing apoptosis. Nuclei were labeled with DAPI. Scale bars, 50μm.

Supplementary Figure 11.

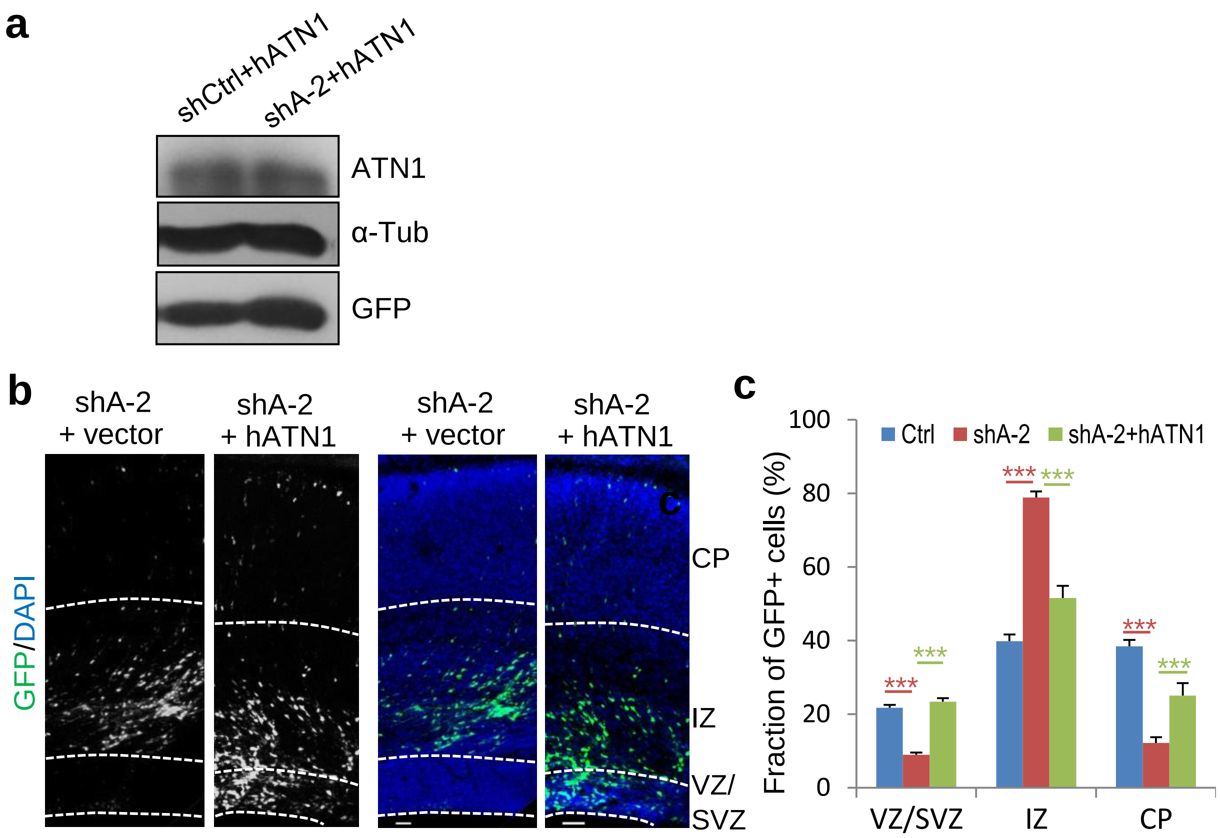

Supplementary Fig. 11. related to Fig. 5. **Overexpression of hATN1 can rescue the ATN1 knockdown induced NPC depletion.**

(a) shA-2 could not knock down human ATN1 (hATN1) expression. hATN1 were transfected into HEK293 cells together with shCtrl or shA-2. 24 hours later, the amount of ATN1 in cell lysates was analyzed by immunoblotting with ATN1 antibody, with α-Tubulin as a loading control and GFP as transfection efficiency control. **(b)** Coronal sections of rat brains electroporated with shA-2 and empty vector or pCAGIG-hATN1 at E16.5 and inspected at E20.5. **(c)** Quantification of cell distribution in (b). Data are means±SEM. ***: $P<0.001$. ShCtrl: n=21/7; shA-2: n=27/9; shA-2+hATN1: n=13/4. One-way ANOVA. n: number of brain slices from different brains.. Nuclei were labeled with DAPI. Scale bars, 50μm.

Supplementary Figure 12.

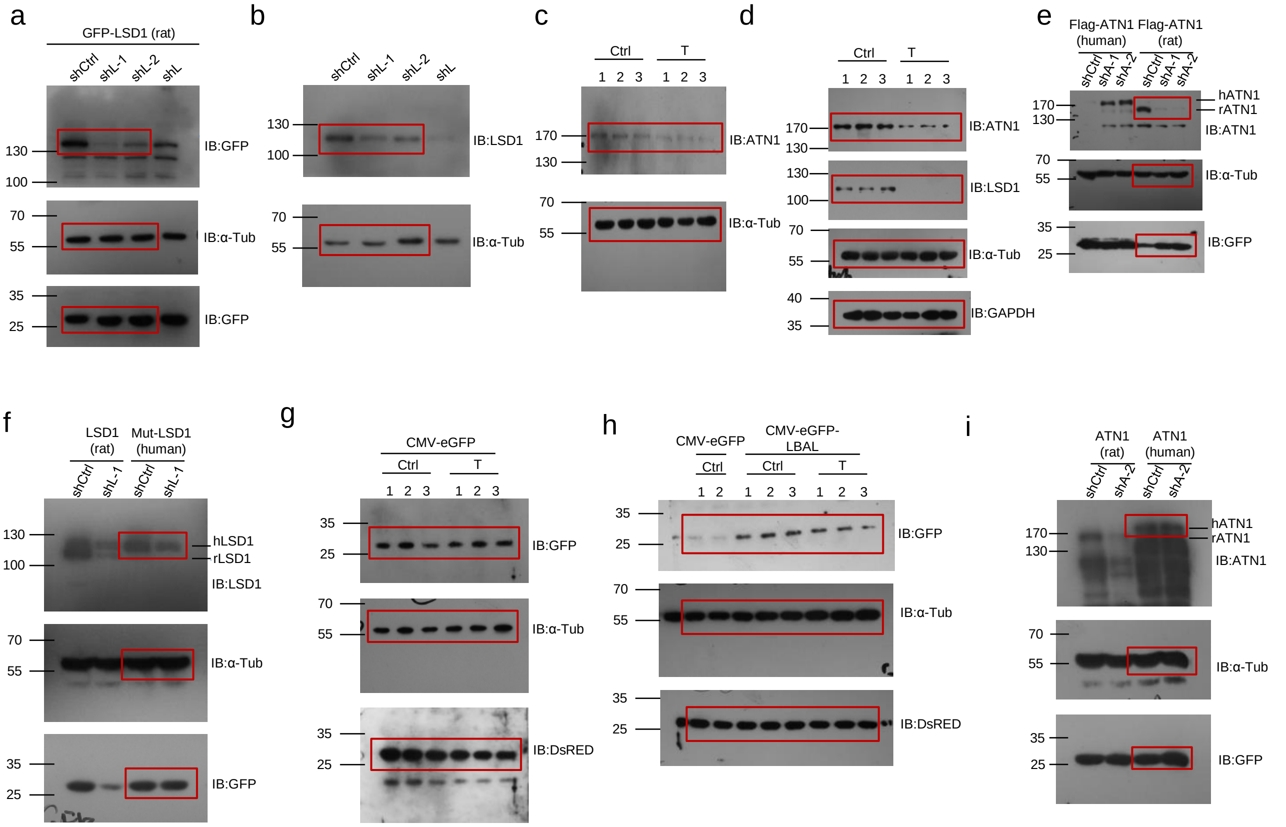

Supplementary Fig. 12. Original WB images. (a) Original WB images of Fig.1b. **(b)** Original WB images of Fig.1c. **(c)** Original WB images of Fig.3f. **(d)** Original WB images of Fig.3h. **(e)** Original WB images of Fig.5c. **(f)** Original WB images of Supplementary Fig. 2c. **(g)** Original WB images of Supplementary Fig. 7b. **(h)** Original WB images of Supplementary Fig. 7c. **(i)** Original WB images of Supplementary Fig. 11a. Molecular weight markers are represented is kDa. IB: immunoblot.

Supplementary Table 1. List of LSD1 binding sites highly enriched in LSD1 ChIP-seq. Genome: rn3.

Peak Region	Length	Summit	Tags	=-10*LOG10 (P value)	Fold of enrichment	Gene ID, Gene Name
chr17:48501822-48502610	789	465	171	2463.45	226.14	NM_001024282, RGD1564767; NM_001107354, Hist1h2an
chr6:75048900-75049947	1048	474	206	2874.68	224.11	NM_001108711, Snx6
chr10:56592043-56594019	1977	716	285	3107.81	220.03	NM_001127658, Amac1
chr2:191037152-191039701	2550	1648	318	2740.6	205.77	NM_001100836, Fcgr1a; NM_001107698, Hist2h3c2; NM_001123469, Hist2h4
chr6:124480637-124481850	1214	616	150	1771.03	162.99	NM_031969, Calm1
chr16:82780192-82781107	916	427	109	1300.52	154.84	NM_001038591, Ing1; NM_001177684, RGD1311612
chr4:161114749-161117223	2475	1043	184	1326.77	150.76	NM_017228, **Atn1**; NM_053908, Ptpn6; NM_139325, Eno2
chr18:71935925-71936667	743	316	98	1215.27	146.69	NM_001173472, RGD1562987; NM_201415, Rpl17
chr6:75051151-75053268	2118	1018	212	1625.05	145.17	NM_001108711, Snx6
chr8:93812117-93813307	1191	630	118	1253.25	144.65	NM_001047916, Syncrip
chr10:90672259-90674051	1793	752	150	1259.29	140.58	NM_001105842, Rdm1

Supplementary Table 2. The sequence of primers used for cloning or Real-time PCR.

Gene or DNA cloned	Primers Sequence for cloning
Human ATN1	5'-catgtcGAATTCatgaagacacgacagaataaagactcg-3' (F)
	5'-acgtcaCTCGAGGCGGCCGCctacagtggcttgtcgctttccttc-3' (R)
Rat ATN1	5'-catgtcGAATTCatgaagacacgacagaataaagactcg-3' (F)
	5'-acgtcaCTCGAGGCGGCCGCctacagcggcttgtcactctccttc-3' (R)
Human LSD1	5'-catgtcGAATTCatgttatctgggaagaaggcggcagccg-3' (F)
	5'-acgtcaGCGGCCGCGGTACCtcacatgcttggggactgctgtgcagg-3' (R)
Rat LSD1	5'-catgtcGAATTCatgttgtctgggaagaaggc-3' (F)
	5'-acgtcaGGTACCtcacatacttggggactgct-3' (R)
LBAL site	5'-CATGTCGAATTCGACAAGGCTCTTCTTGGTCTTGGAATCTC-3' (F)
	5'-ACGTCATCTAGATCAAGAGTCAGAACATTTTAGGCCGGGC-3' (R)
**Gene or DNA**	**Primers Sequence for Real-time PCR**
rat *β-actin*	5'-AGGGAAATCGTGCGTGAC-3' (F)
	5'-GATAGTGATGACCTGACCGT-3' (R)
rat *ATN1*	5'-TATAGCCAAGCAGGTCCCAATGGT-3' (F)
	5'-TGAGCATGAATAGGCGGCTTGAGA-3' (R)
mouse *GAPDH*	5'-TGATGACATCAAGAAGGTGGTGAAG-3' (F)
	5'-TCCTTGGAGGCCATGTAGGCCAT-3' (R)
mouse *ATN1*	5'-TATAACCAAGCAGGTCCCAATGGT-3' (F)
	5'-TGGGATGAAGAGGGGTGTGAACAT-3' (R)
rat LBAL	5'-CAGACTAGGCTCCTCACCCT-3' (F)
	5'-GCTTTCGGCCCACTCTAGTT-3' (R)
mouse LBAL	5'-CAGACTAGGCTCTTTCCTCACCCT-3' (F)
	5'-GGCTTTGATCCTTCTCTGGTT-3' (R)
